# Behavioral Reaction and *c-fos* Expression after Opioids Injection into the Pedunculopontine Tegmental Nucleus and Electrical Stimulation of the Ventral Tegmental Area

**DOI:** 10.3390/ijms24010512

**Published:** 2022-12-28

**Authors:** Grażyna Jerzemowska, Karolina Plucińska, Aleksandra Piwka, Magdalena Podlacha, Jolanta Orzeł-Gryglewska

**Affiliations:** 1 Department of Animal and Human Physiology, Faculty of Biology, University of Gdansk, 59 Wita Stwosza Str., 80-308 Gdansk, Poland; 2 Department of Molecular Biology, Faculty of Biology, University of Gdansk, 59 Wita Stwosza Str., 80-308 Gdansk, Poland

**Keywords:** morphine, naloxone, dopamine, ventral tegmental area, pedunculopontine tegmental nucleus, opioid receptors

## Abstract

The pedunculopontine tegmental nucleus (PPN) regulates the activity of dopaminergic cells in the ventral tegmental area (VTA). In this study, the role of opioid receptors (OR) in the PPN on motivated behaviors was investigated by using a model of feeding induced by electrical VTA-stimulation (Es-VTA) in rats (male Wistar; n = 91). We found that the OR excitation by morphine and their blocking by naloxone within the PPN caused a change in the analyzed motivational behavior and neuronal activation. The opioid injections into the PPN resulted in a marked, dose-dependent increase/decrease in latency to feeding response (FR), which corresponded with increased neuronal activity (c-Fos protein), in most of the analyzed brain structures. Morphine dosed at 1.25/1.5 µg into the PPN significantly reduced behavior induced by Es-VTA, whereas morphine dosed at 0.25/0.5 µg into the PPN did not affect this behavior. The opposite effect was observed after the naloxone injection into the PPN, where its lowest doses of 2.5/5.0 μg shortened the FR latency. However, its highest dose of 25.0 μg into the PPN nucleus did not cause FR latency changes. In conclusion, the level of OR arousal in the PPN can modulate the activity of the reward system.

## 1. Introduction

Evolutionarily, the mesolimbic system (ML) is the older part of the brain that connects the motor subcortex structures with the limbic system [1]. The ML controls and processes information related to emotional, cognitive, and sensory inputs, which are then delivered mainly to the phylogenetically younger areas of the brain [1,2]. Moreover, ML is responsible for regulating behavior related to motivation and reward [1,3]. Thus, this system enables complex hierarchical control of sexual, exploratory, and food-related behaviors, among others [2], which has been shown through experiments in which electrical stimulation of a critical ML structure—the ventral tegmental area (VTA)—induces food and exploratory reactions [4].

It is known that dopamine (DA) is widely implicated in the control of feeding [5]. Widespread disruption of dopaminergic neuronal function caused by chemical lesions results in severe aphagia [5,6]. However, the process of firing inhibition through optogenetic stimulation of VTA GABAergic neurons (GABA) is temporally precise and leads to the disrupted licking of solutions made out of sucrose in hungry mice [5,7]. Correspondingly, sated animals, which are attenuated by DA receptor antagonists at low doses that preserve locomotor function, experience current stimulation–induced eating [8,9]. Although DA plays a crucial role in the initiation of eating, its ability to promote eating without physiological needs is unclear [5].

It is known that the most important ML structures are the VTA and nucleus accumbens (Acb) [10]. The primary synaptic mediators in this system are DA, glutamate (Glu), and gamma-aminobutyric acid (GABA) [11,12]. The neural projections from the VTA to the Acb are known to have a critical role in the development of reward-related behaviors [13]. The dopaminergic-VTA neurons also innervate several regions of the prefrontal cortex (PFC), amygdala (Amg) and hippocampus (Hip), lateral habenula (LHb), and other structures. Moreover, these structures have glutamatergic interconnections with each other and with the Acb [14].

The binding of opiates primarily drives addiction to G-coupled opiate receptors (OR) such as the µ-opioid receptor (MOR). Opiate binding leads to activation of the receptors and associated G proteins tethered to the cells’ receptors (the activation of the latter is aided by the action of second messengers such as cyclic AMP, or cAMP). The G proteins transduce signals from the receptors to activate intra-cellular signaling pathways through the phosphorylation of effector molecules such as protein kinases and induction of other signaling molecules. These signaling events lead to functional changes in a cell (changes in the expression levels of genes, including the *c-fos* proto-oncogene), which mediate the processes of addiction to opiates [15]. 

Regarding morphine, there is substantiated experimental evidence that it influences the phosphorylation of G-protein coupled receptors (GPCR) and the binding of regulatory proteins [16]. Therefore, these changes can lead to modification in the synthesis of target genes, which may be responsible for opiate-induced long-lasting neural plasticity [17]. In the ML system, the response of DA cells located in the VTA to opioids is evoked from the presynaptic opioid inhibition of GABA, which leads to an increase in DA neurons activity. Thereupon, an augmentation in the DA secretion in the Acb can be spotted, leading to a reward response [17,18,19].

It is also known that Acb is the entrance structure to the basal ganglia system [20]. At the same time, the pedunculopontine tegmental nucleus (PPN) is the exit station through which information from Acb passes back, coupling fields related to emotions, anxiety, and areas associated with motor function [21,22]. It is also possible that OR in the PPN perceives anxiety-related stimuli, enhancing anxiety reactions [23]. Moreover, changes in the activation of the PPN can significantly affect the excitability of DA cells in the midbrain through its direct connections [24,25,26]. Therefore, it is essential to determine the interdependence between the PPN and VTA and their influence on other brain structures in opioid addiction.

Our previous studies demonstrated that the PPN and VTA and their complex interhemispheric relationship are located in the same central circuitry regulating motivational behavior [4,27]. It has been found that the PPN is involved in motivational behavior by changing the activation of major neuroreceptors, such as cholinergic, GABAergic [4], or glutamatergic [27] receptors. However, the mechanisms of the formation of behavioral reactions such as food and exploratory or pathological reactions appearing during addiction, e.g., to opiates, are not fully understood. Therefore, our work aimed to investigate the role of OR activation in the PPN nucleus by using a model of feeding response (FR) induced by unilateral electrical VTA-stimulation (Es-VTA) in rats.

## 2. Results

### 2.1. Behavioral Analysis

#### 2.1.1. Morphine

**Figure 1:**
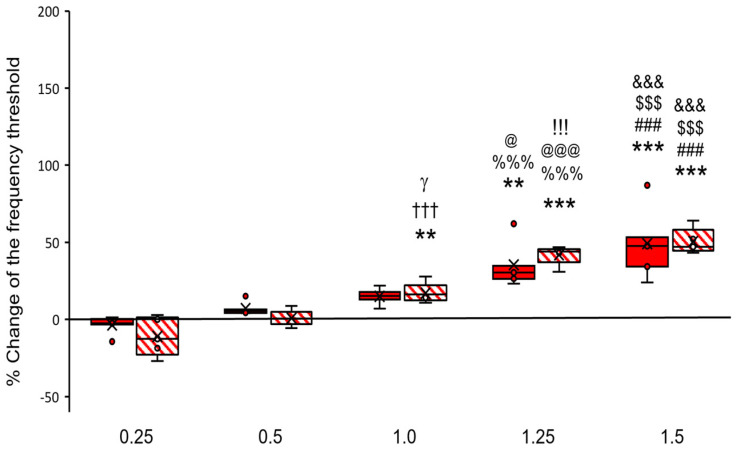
Percent change of the frequency threshold in the feeding response (FR) during unilateral electrical VTA stimulation (Es-VTA) directly after morphine injection at different doses (0.25 µg/0.5 µL, 0.5 µg/0.5 µL, 1.0 µg/0.5 µL, 1.25 µg/0.5 µL, or 1.5 µg/0.5 µL) into the PPN in the contralateral (used for each dose n = 5) (red-filled bars) and ipsilateral hemisphere (used for each dose n = 5) (red-hatched bars) compared to artificial cerebrospinal fluid (ACSF; a value of 0.0% on the y-axis) injected in the PPN as baseline control. Tukey’s post hoc test: ** *p* < 0.01, *** *p* < 0.00 directly above the bars indicate a significant difference from experimental groups with different injection doses (1.0, 1.25, and 1.5 µg) compared to baseline (Mc, Mi groups vs. baseline); ^###^ *p* < 0.001 indicate a significant difference from morphine injection doses (dose 1.5 µg vs. dose 0.25 µg) in the experimental groups (Mc, Mi groups); ^$$$^ *p* < 0.001 indicate a significant difference from morphine injection doses (dose 1.5 µg vs. dose 0.5 µg) in the experimental groups (Mc, Mi groups); ^&&&^ *p* < 0.001 indicate a significant difference from morphine injection doses (dose 1.5 µg vs. dose 1.0 µg) in the experimental groups (Mc, Mi groups); ^%%%^ *p* < 0.001 indicate a significant difference from morphine injection doses (dose 1.25 µg vs. dose 0.25 µg) in the experimental groups (Mc, Mi groups); ^@^ *p* < 0.05, ^@@@^ *p* < 0.001 indicates a significant difference from morphine injection doses (dose 1.25 µg vs. dose 0.5 µg) in the experimental groups (Mc, Mi groups); !!! *p* < 0.001 indicate a significant difference from morphine injection doses (dose 1.25 µg vs. dose 1.0 µg) in the experimental group (Mi groups); ^†††^ *p* < 0.001 indicate a significant difference from morphine injection doses (dose 1.0 µg vs. dose 0.25 µg) in the experimental group (Mi group); ^γ^ *p* < 0.05 indicate a significant difference from morphine injection doses (dose 1.0 µg vs. dose 0.5 µg) in the experimental group (Mi group).

Analysis (two-way ANOVA) showed that microinjection of morphine into the PPN reduced the FR induced by Es-VTA depending on the doses of morphine only (F_(5,48)_ = 50.434, *p* < 0.001), but not on the hemisphere of the injection (F_(1,48)_ = 0.674, *p* > 0.05). The different morphine doses, which were injected into the PPN, reduced the Es-VTA FR-induced to varying percentage degrees both in the contralateral group (Mc-1, Mc-2, Mc-3, Mc-4, and Mc-5 vs. baseline, F_(5,29)_ = 14.527, *p* < 0.001) and in the ipsilateral group (Mi-1, Mi-2, Mc-3, Mc-4, and Mc-5 vs. baseline, F_(5,29)_ = 55.779, *p* < 0.001). Tukey’s post hoc test validated that these reactions were observed only at three (1.0, 1.25, and 1.5 µg) of the five doses (0.25, 0.5, 1.0, 1.25, and 1.5 µg) with the strongest effect observed at the two highest doses (1.25 and 1.5 µg) in rats from the Mc groups (baseline, 0.0% vs. Mc-1, −3.71 ± 2.79%, *p* > 0.05; Mc-2, 7.10 ± 2.03%, *p* > 0.05; Mc-3, 14.92 ± 2.51%, *p* > 0.05; Mc-4, 35.38 ± 6.96%, *p* < 0.01; and Mc-5, 49.25 ± 10.72%, *p* < 0.001) (Figure 1, red-filled bars as Mc groups and baseline as 0.0% value on the y-axis) and in rats from the Mi groups (baseline, 0.0% vs. Mi-1, −11.05 ± 5.60, *p* > 0.05; Mi-2, 0.87 ± 2.32, *p* > 0.05; Mi-3, 17.08 ± 2.89, *p* < 0.01; Mi-4, 41.89 ± 2.78, *p* < 0.001; and Mi-5, 50.54 ± 3.67, *p* < 0.001) (Figure 1, red-hatched bars as Mi groups and baseline as 0.0% value on the y-axis). Moreover, there were differences between the experimental groups. This effect was noticeable for both rats in the Mc groups (Mc-5 vs. Mc-1, *p* < 0.001; Mc-5 vs. Mc-2, *p* < 0.001; Mc-5 vs. Mc-3, *p* < 0.001; Mc-4 vs. Mc-1, *p* < 0.001; and Mc-4 vs. Mc-2, *p* < 0.05) (Figure 1, red-filled bars) and in the Mi groups (Mi-5 vs. Mi-1, *p* < 0.001; Mi-5 vs. Mi-2, *p* < 0.001; Mi-5 vs. Mi-3, *p* < 0.001; Mi-4 vs. Mi-1, *p* < 0.001; Mi-4 vs. Mi-2, *p* < 0.001; Mi-4 vs. Mi-3, *p* < 0.001; Mi-3 vs. Mi-1, *p* < 0.001; and Mi-3 vs. Mi-2, *p* < 0.05) (Figure 1, red-hatched bars).

**Figure 2:**
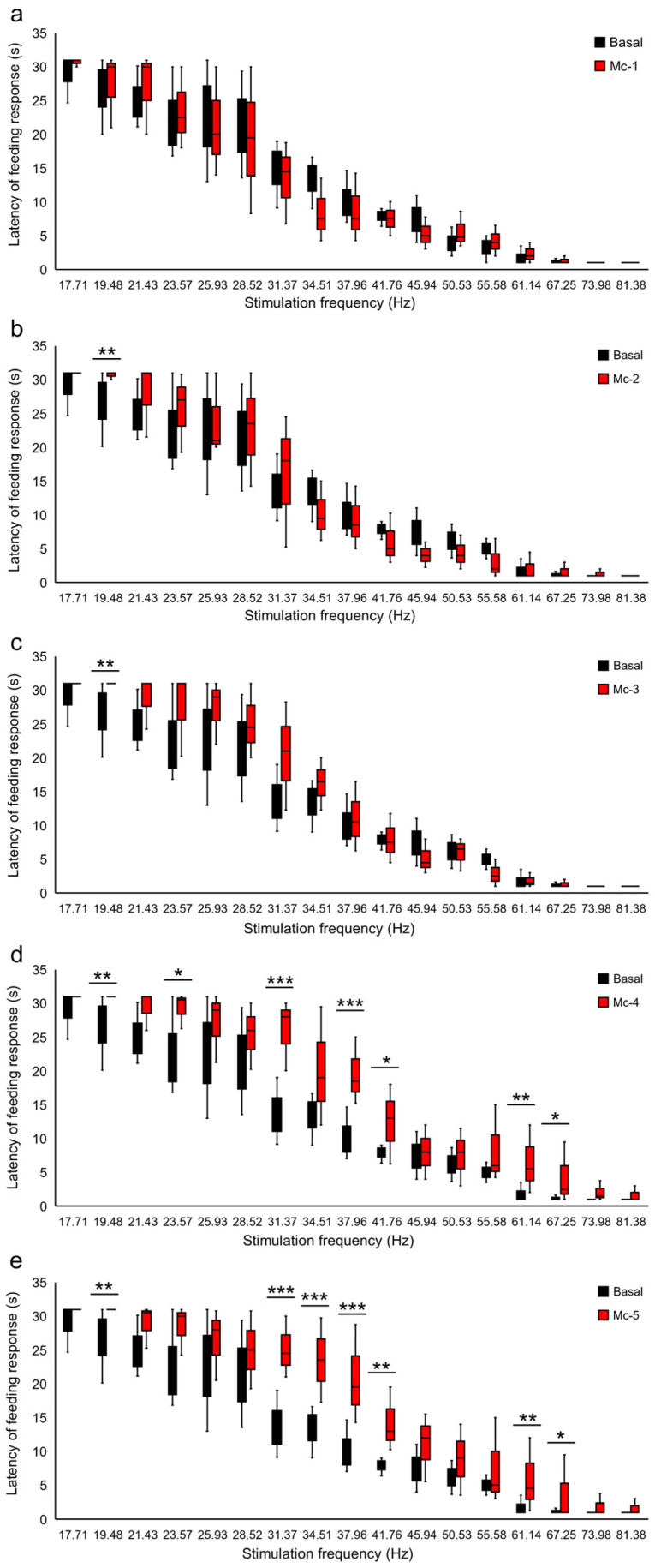
Destabilization of the VTA electrical stimulation (Es-VTA)-induced feeding response (FR) directly after morphine administration at different doses [0.25 µg/0.5 µL (Mc-1 group) (**a**), 0.5 µg/0.5 µL (Mc-2 group) (**b**), 1.0 µg/0.5 µL (Mc-3 group) (**c**), 1.25 µg/0.5 µL (Mc-4 group) (**d**), and 1.5 µg/0.5 µL (Mc-5 group) (**e**)] injected into the PPN in the contralateral hemisphere (red-filled bars) (n = 5 for each dose) compared to artificial cerebrospinal fluid injection (ACSF; 0.5 µL) into the PPN as basal groups (black-filled bars) (**a**–**e**). * *p* < 0.05, ** *p* < 0.01, and *** *p* < 0.001 (Tukey’s post hoc test) indicate significant differences in the FR latency at different frequencies of Es-VTA in the Mc-1, Mc-2, Mc-3, Mc-4, and Mc-5 experimental groups compared to the basal group.

**Figure 3:**
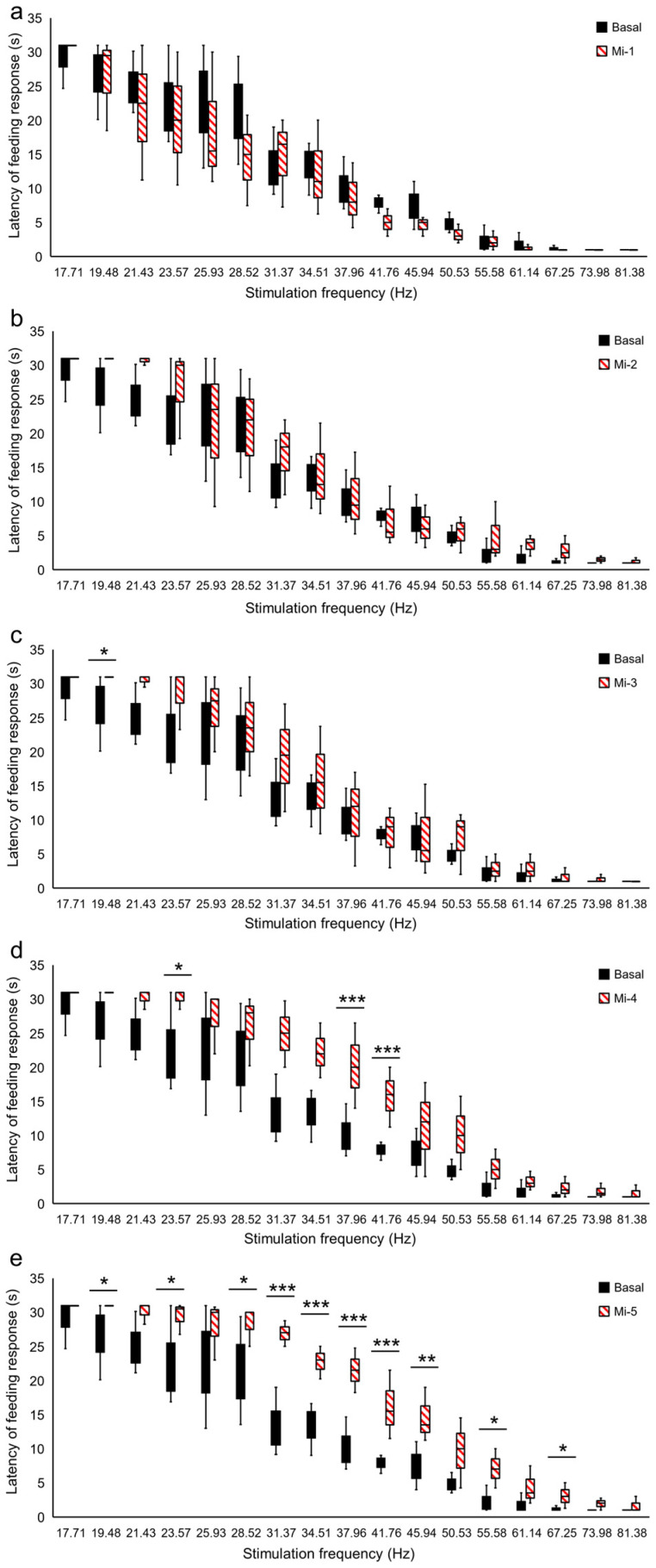
Destabilization of the VTA electrical stimulation (Es-VTA)-induced feeding response (FR) directly after morphine injection at different doses [0.25 µg/0.5 µL (Mi-1 group) (**a**) 0.5 µg/0.5 µL (Mi-2 group) (**b**), 1.0 µg/0.5 µL (Mi-3 group) (**c**), 1.25 µg/0.5 µL (Mi-4 group) (**d**), and 1.5 µg/0.5 µL (Mi-5 group) (**e**)] injected into the PPN in the ipsilateral hemisphere (red-hatched bars) (n = 5 for each dose) compared to artificial cerebrospinal fluid injection (ACSF; 0.5 µL) into the PPN as basal groups (black-filled bars) (**a**–**e**). * *p* < 0.05, ** *p* < 0.01, and *** *p* < 0.001 (Tukey’s post hoc test) indicate significant differences in the FR latency at different frequencies of Es-VTA in the Mi-1, Mi-2, Mi-3, Mi-4, and Mi-5 experimental groups compared to the basal group.

The frequency threshold was elevated for the FR induced by Es-VTA, which was accompanied by a rightward shift of the relationship of latency–stimulation frequency, both in rats from the Mc groups (Figure 2) and the Mi groups (Figure 3). The FR induced by Es-VTA latency analysis after injection of morphine showed an interaction between the dose of morphine and the frequency of the stimulation current (F_(85,1932)_ = 2.045, *p* <0.001) in rats in the Mc groups. In addition, there was a statistical difference in the latency of this response between the current frequencies (from 17.71 Hz to 81.38 Hz) (F_(17,1932)_ = 403.607, *p* < 0.001) and injection doses used (F_(5,1932)_ = 32.804, *p* < 0.001) in the PPN in the contralateral hemisphere (Mc-1, Mc-2, Mc-3, Mc-4, Mc-5, and baseline). In rats in the Mc groups (Figure 2a–e), the curve shifts after morphine injection (red-filled bars) in comparison to the baseline (ACSF injection) (black-filled bars) were significant only at a single frequency (19.48 Hz) in the groups with medium doses of morphine injection (Mc-2, dose 0.5 µg, *p* < 0.01) (Figure 2b) (Mc-3, dose 1.0 µg, *p* < 0.01) (Figure 2c). In the case of the highest morphine doses (Mc-4, dose 1.25 µg and Mc-5, dose 1.5 µg) (Figure 2d,e; red-filled bars), the prolonged latency of FR induced by Es-VTA in comparison to the baseline (Figure 2d,e; black-filled bars) was observed at the following frequencies: 19.48 Hz (Mc-4, *p* < 0.01; Mc-5, *p* < 0.01), 23.57 Hz (Mc-4, *p* < 0.05), 31.37 Hz (Mc-4, *p* < 0.001; Mc-5, *p* < 0.001), 34.51 Hz (Mc-5, *p* < 0.001), 37.96 Hz (Mc-4, *p* < 0.001; Mc-5, *p* < 0.001), 41.76 Hz (Mc-4, *p* < 0.05; Mc-5, *p* < 0.01), 61.14 Hz (Mc-4, *p* < 0.01; Mc-5, *p* < 0.01), and 67.25 Hz (Mc-4, *p* < 0.05; Mc-5, *p* < 0.05). 

The latency of the FR during Es-VTA showed an interaction between the dose of morphine in the PPN and the frequency of the stimulation current (F_(85,1932)_ = 2.913, *p* < 0.001) in rats from the Mi groups. There was also a significant difference in the current frequencies (from 17.71 Hz to 81.38 Hz) (F_(17,1932)_ = 419.991, *p* < 0.001) and the injection doses used (F_(5,1932)_ = 53.043, *p* < 0.001) in the PPN in the ipsilateral hemisphere (Mc-1, Mc-2, Mc-3, Mc-4, Mc-5, and baseline). In rats from the Mi groups (Figure 3a–e), the prolonged latency of reaction was also observed in the groups with the highest doses of morphine (Mi-3: dose 1.0 µg, Mi-4: dose 1.25 µg, and Mi-5: dose 1.5 µg) (Figure 3c–e; red-hatched bars) in comparison to the baseline (Figure 3c–e; black-filled bars) at the following frequencies: 19.48 Hz (Mi-3, *p* < 0.05; Mi-4, *p* < 0.05; Mi-5, *p* < 0.05), 23.57 Hz (Mi-4, *p* < 0.05; Mi-5, *p* < 0.05), 28.52 Hz (Mi-5, *p* < 0.05), 31.37 Hz (Mi-4, *p* < 0.001; Mi-5, *p* < 0.001), 34.51 Hz (Mi-4, *p* < 0.001; Mi-5, *p* < 0.001), 37.96 Hz (Mi-4, *p* < 0.001; Mi-5, *p* < 0.001), 41.76 Hz (Mi-4, *p* < 0.001; Mi-5, *p* < 0.001), 45.94 Hz (Mi-5, *p* < 0.01), 55.58 Hz (Mi-5, *p* < 0.05), and 67.25 Hz (Mi-5, *p* < 0.05). 

#### 2.1.2. Naloxone

**Figure 4:**
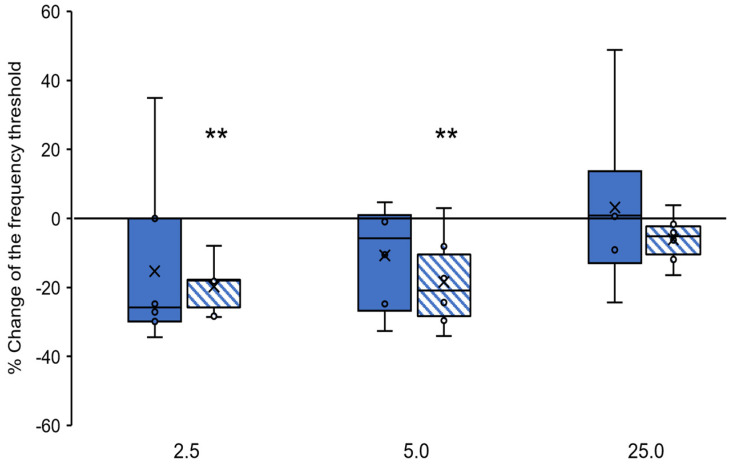
Percent change of the frequency threshold in the feeding response (FR) during unilateral electrical VTA stimulation (Es-VTA) directly after different doses of naloxone (2.5 µg/0.5 µL, 5.0 µg/0.5 µL, or 25.0 µg/0.5 µL) injected into the PPN in the contralateral (blue-filled bars) and ipsilateral hemisphere (blue-hatched bars) (n = 6 for each dose) compared to artificial cerebrospinal fluid (ACSF; a value of 0.0% on the y-axis) injection into the PPN as baseline control. Tukey’s post hoc test: ** *p* < 0.01 indicates a significant difference between the experimental groups (2.5 and 5.0 µg doses) compared to the baseline (Ni groups vs. baseline).

In naloxone-injected rats, there was no interaction between the drug dose and the hemisphere of the injection (F_(3,40)_ = 0.488, *p* > 0.05). However, there was variation only between naloxone doses (F_(3,40)_ = 6.704, *p* < 0.001), but not between the injection hemisphere (F_(1,40)_ = 0.978, *p* > 0.05). Subsequent analysis (Tukey post hoc test) showed that in rats from the Ni groups, the single naloxone injection into the PPN caused a percent reduction in the threshold of the uFR induced by Es-VTA, but only at the lowest doses (2.5 µg, Ni-1, −19.77 ± 3.16 and 5.0 µg, Ni-2, −18.41 ± 5.69) (Figure 4, blue-hatched bars) compared to the control (*p* < 0.01) (Figure 4, value of 0.0% on the y-axis). At the highest naloxone dose (25.0 µg, Ni-3, −6.09 ± 2.95), we found no change in the threshold of FR induced by Es-VTA (*p* > 0.05) (Figure 4). In rats in the Nc groups, which were injected with different single naloxone doses into the PPN (2.5 µg, Nc-1, −21.35 ± 7.21; 5.0 µg, Nc-2, −10.75 ± 6.11; and 25.0 µg, Nc-3, 3.17 ± 19.01) (Figure 4, blue-filled bars), there was no percent change in the FR induced by Es-VTA in comparison to the baseline (*p* > 0.05) (Figure 4, value of 0.0% on the y-axis). 

**Figure 5:**
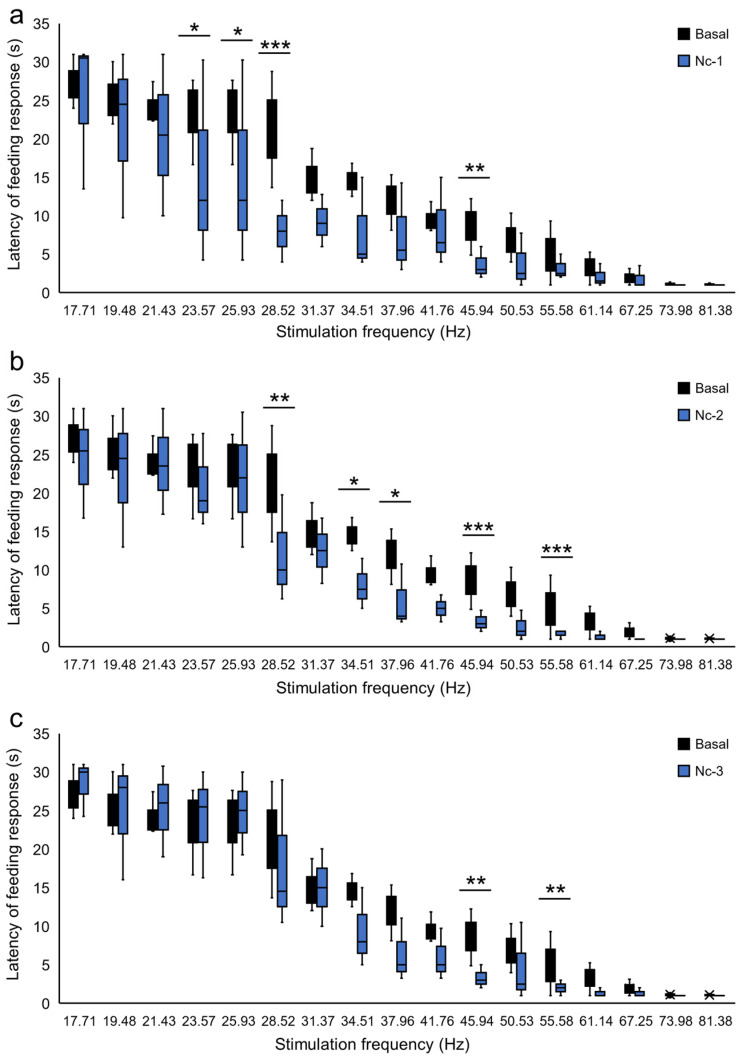
Destabilization of the VTA electrical stimulation (Es-VTA)-induced feeding response (FR) directly after different naloxone doses [2.5 µg/0.5 µL (Nc-1 group) (**a**), 5.0 µg/0.5 µL (Nc-2 group) (**b**), and 25.0 µg/0.5 µL (Nc-3 group) (**c**)] injected into the PPN in the contralateral hemisphere (blue filled bars) (n = 6 for each dose) compared to artificial cerebrospinal fluid injection (ACSF; 0.5 µL) into the PPN as basal groups (black filled bars). * *p* < 0.05, ** *p* < 0.01, and *** *p* < 0.001 (Tukey’s post hoc test) indicate significant differences in the FR latency at different frequencies of Es-VTA in the Nc-1, Nc-2, and Nc-3 experimental groups compared to the basal group.

**Figure 6:**
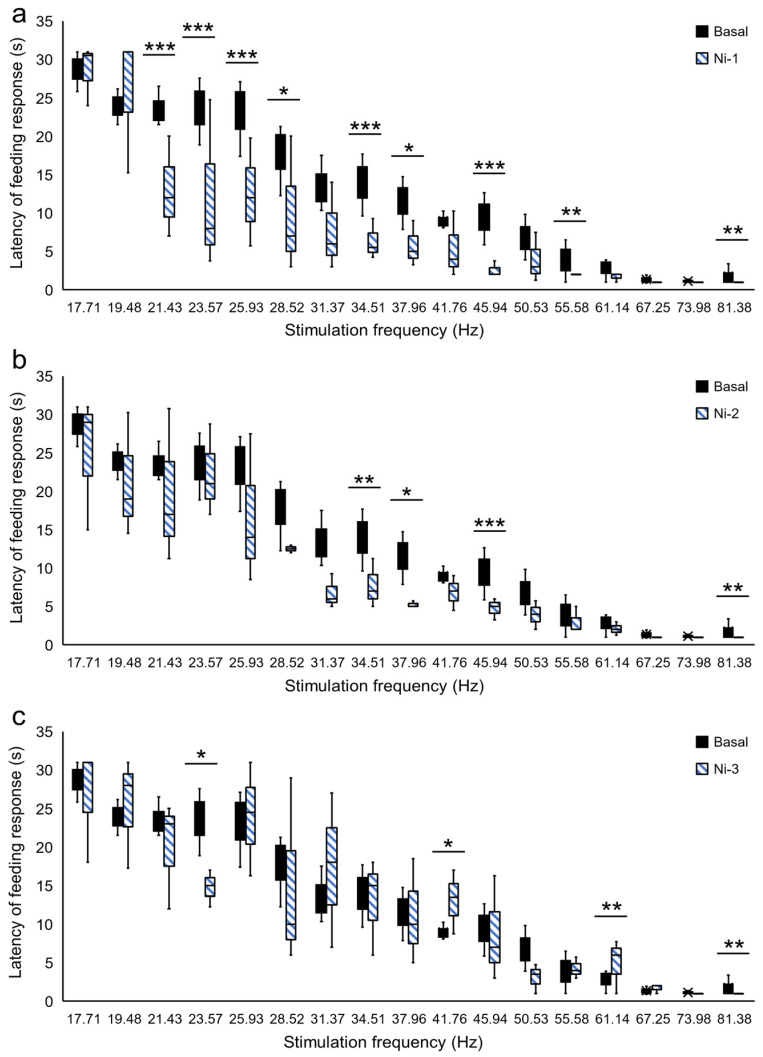
Destabilization of the VTA electrical stimulation (Es-VTA)-induced feeding response (FR) directly after different naloxone doses [2.5 µg/0.5 µL (Ni-1 group) (**a**), 5.0 µg/0.5 µL (Ni-2 group) (**b**), and 25.0 µg/0.5 µL (Ni-3 group) (**c**)] injected into the PPN in the ipsilateral hemisphere (blue hatched bars) (n = 6 for each dose) compared to artificial cerebrospinal fluid (ACSF; 0.5 µL) injected into the PPN as basal groups (black filled bars). * p < 0.05, ** *p* < 0.01, and *** *p* < 0.001 (Tukey’s post hoc test) indicate significant differences in the FR latency at different frequencies of Es-VTA in the Ni-1, Ni-2, and Ni-3 experimental groups compared to the basal group.

In rats in the Nc group, analysis of the FR latency during Es-VTA and naloxone injection into the PPN showed an interaction between the naloxone dose and the frequency of the stimulation current (F_(48,1544)_ = 1.456, *p* < 0.05). Moreover, there was a statistical difference in the latency of this response within the applied current frequencies during this stimulation (from 17.71 Hz to 81.38 Hz) (F_(16,1544)_ = 168.076, *p* < 0.001) and the single naloxone injection doses (F_(3,1544)_ = 26.909, *p* < 0.001) in rats in the Nc groups (Nc-1, Nc-2, Nc-3, and baseline). A similar result was found in the analysis of the FR latency induced by Es-VTA after PPN naloxone injection in rats in the Ni groups, which showed a statistical dependence of the naloxone dose on the frequency of the stimulation current (F_(48,1544)_ = 3.093, *p* < 0.001). Moreover, a significant difference was found in the current frequencies (from 17.71 Hz to 81.38 Hz) (*p* < 0.001) and in the applied PPN injection doses (*p* < 0.001) (Ni-1, Ni-2, Ni-3, and baseline). Figure 5 and Figure 6 show that the lowest doses of naloxone (2.5 µg in the Nc-1 and Ni-1 groups and 5.0 µg in the Nc-2 and Ni-2 groups) injected into the PPN contra- (Figure 5a–c) (blue-filled bars) and ipsilateral hemispheres (Figure 6a–c) (blue-hatched bars) during unilateral Es-VTA decreased the latency to initiate the FR in comparison to the baseline (Figure 5a–c and Figure 6a–c) (black-filled bars). Significant shortening of the FR latency during Es-VTA occurred in rats with naloxone injected into the ipsilateral PPN at the lowest dose (2.5 µg) (Ni-1 group), which was observed during most of the tested frequencies: 21.43 Hz to 25.93 Hz (*p* < 0.001), 28.52 Hz (*p* < 0.05), 34.51 Hz (*p* < 0.001), 37.96 Hz (*p* < 0.05), 45.94 Hz (*p* < 0.001), 55.58 Hz (*p* < 0.01), and 81.38 Hz (*p* < 0.01) (Figure 6a).

### 2.2. Immunofluorescent Detection of c-Fos Protein in Select Brain Structures

Rat brains were the only subject of performance of the immunofluorescence analysis of c-Fos+ cell nuclei with the highest dosage of morphine (1.5 µg: Mc-5 group, n = 5 and Mi-5 group, n = 5) and naloxone (25.0 µg: Nc-3 group, n = 6 and Ni-3 group, n = 6) administered into the PPN immediately before Es-VTA. The criteria-eligible control brains came from rats that received the 14-day Es-VTA (the total number of days of Es-VTA from both the screening and proper Es-VTA procedures in the rats of the experimental groups, but without injection procedure) (baseline group, n = 5).

Due to the lack of significant interhemispheric differences in any brain structure (after the completion of the one-way ANOVA analysis, which included the brain hemisphere left or right as a factor, data not presented), the data collected have been considered as averaged, yielding one number for each of the tested brain regions.

**Figure 7:**
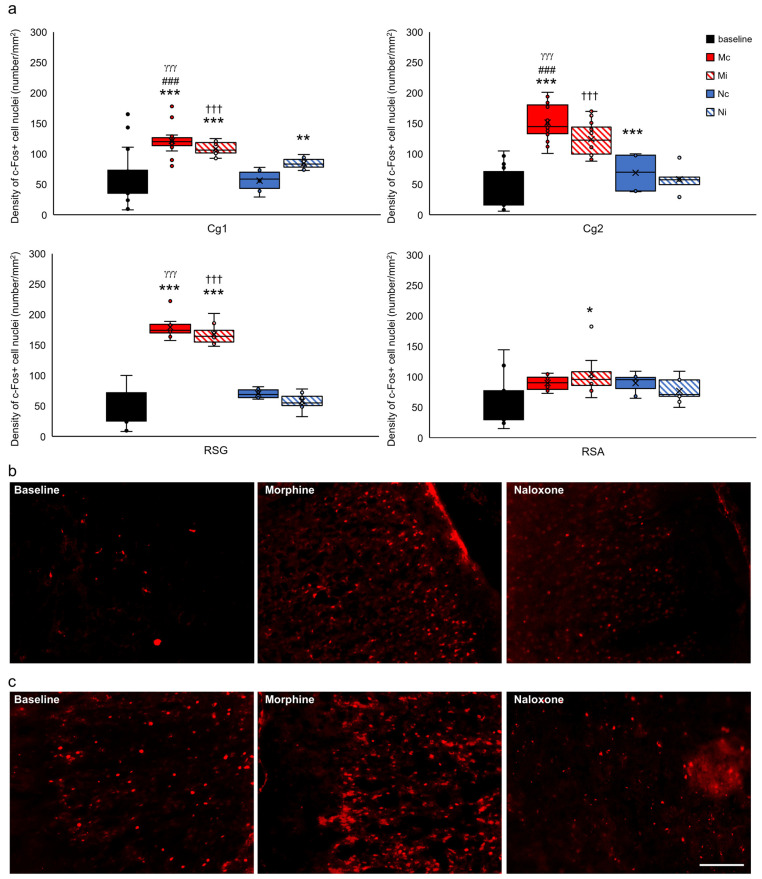
Density of c-Fos+ cell nuclei (number/1 mm^2^) in select limbic structures of the cortex in the brains of rats that were subjected to VTA electrical stimulation (Es-VTA) only (baseline group, n = 5) (black-filled bars) and in the brains of rats administered Es-VTA and a one-time injection of morphine (1.5 µg) or naloxone (25.0 µg) injected into the PPN in the contralateral (Mc, n = 5, red-filled bars; Nc, n = 6, blue-filled bars) and ipsilateral hemispheres (Mi, n = 5, red-hatched bars; Ni, n = 6, blue-hatched bars) (**a**). Representative photomicrographs of c-Fos-labeled neurons in the Cg1 (**b**) and Cg2 (**c**) structures are shown. Scale bar = 100 µm (white line: right lower corner of the last photo, panel (**c**)). Tukey’s post hoc test: * *p* < 0.05, ** *p* < 0.01, and *** *p* < 0.001 indicate a significant difference between the baseline and other experimental groups (Mc, Mi, Nc, and Ni groups); ^###^ *p* < 0.001 indicate a significant difference in the morphine groups between different injection site: contra- or ipsilateral hemispheres (Mc vs. Mi group); ^γγγ^ *p* < 0.001 indicate a significant difference in the contralateral side groups between different injection drug: morphine or naloxone (Mc vs. Nc group); ^†††^ *p* < 0.001 indicate a significant difference in the ipsilateral side groups between different injection drug: morphine or naloxone (Mi vs. Ni group).

**Figure 8:**
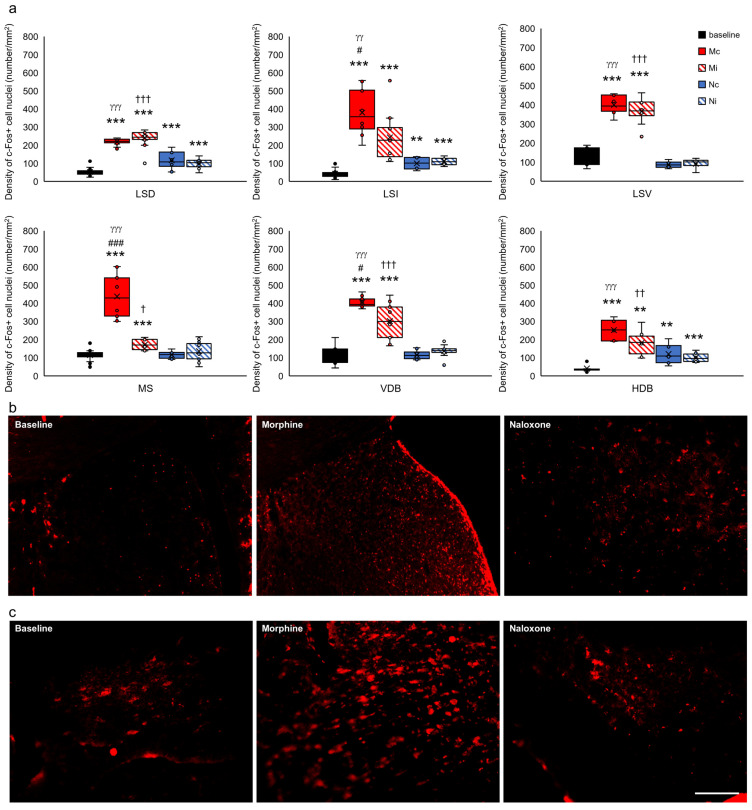
Density of c-Fos+ cell nuclei (number/1 mm^2^) in select structures of the septal system in the brains of rats that were subjected to VTA electrical stimulation (Es-VTA) only (baseline group, n = 5) (black-filled bars) and in the brains of rats administered Es-VTA and a one-time injection of morphine (1.5 µg) or naloxone (25.0 µg) injected into the PPN in the contralateral (Mc, n = 5, red-filled bars; Nc, n = 6, blue-filled bars) and ipsilateral hemispheres (Mi, n = 5, red-hatched bars; Ni, n = 6, blue-hatched bars) (**a**). Representative photomicrographs of c-Fos-labeled neurons in the LSD (**b**) and VDB (**c**) structures are shown. Scale bar = 100 µm (white line: right lower corner of the last photo, panel (**c**)). Tukey’s post hoc test: ** *p* < 0.01, and *** *p* < 0.001 indicate a significant difference between the baseline and other experimental groups (Mc, Mi, Nc, and Ni groups); ^#^ *p* < 0.05, ^###^ *p* < 0.001 indicate a significant difference in the morphine groups between different injection site: contra- or ipsilateral hemispheres (Mc vs. Mi group); ^γγ^ *p* < 0.01, ^γγγ^ *p* < 0.001 indicate a significant difference in the contralateral side groups between different injection drug: morphine or naloxone (Mc vs. Nc group); ^†^ *p* < 0.05, ^††^ *p* < 0.01, ^†††^ *p* < 0.001 indicate a significant difference in the ipsilateral side groups between different injection drug: morphine or naloxone (Mi vs. Ni group).

**Figure 9:**
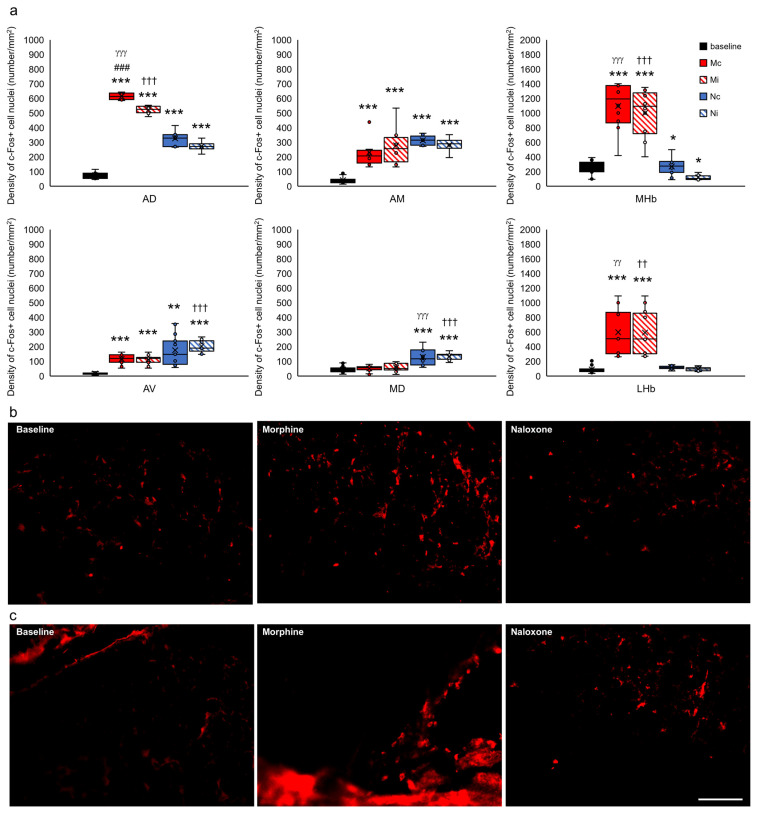
Density of c-Fos+ cell nuclei (number/1 mm^2^) in select limbic structures of the thalamus in the brains of rats that were subjected to VTA electrical stimulation (Es-VTA) only (baseline group, n = 5) (black-filled bars) and in the brains of rats administered Es-VTA and a one-time injection of morphine (1.5 µg) or naloxone (25.0 µg) injected into the PPN in the contralateral (Mc, n = 5, red-filled bars; Nc, n = 6, blue-filled bars) and ipsilateral hemispheres (Mi, n = 5, red-hatched bars; Ni, n = 6, blue-hatched bars) (**a**). Representative photomicrographs of c-Fos-labeled neurons in the LHb (**b**) and MHb (**c**) structures are shown. Scale bar = 100 µm (white line: right lower corner of the last photo, panel (**c**)). Tukey’s post hoc test: * *p* < 0.05, ** *p* < 0.01, and *** *p* < 0.001 indicate a significant difference between the baseline and other experimental groups (Mc, Mi, Nc, and Ni groups); ^###^ *p* < 0.001 indicate a significant difference in the morphine groups between different injection site: contra- or ipsilateral hemispheres (Mc vs. Mi group); ^γγ^ *p* < 0.01, ^γγγ^ *p* < 0.001 indicate a significant difference in the contralateral side groups between different injection drug: morphine or naloxone (Mc vs. Nc group); ^††^ *p* < 0.01, ^†††^ *p* < 0.001 indicate a significant difference in the ipsilateral side groups between different injection drug: morphine or naloxone (Mi vs. Ni group).

**Figure 10:**
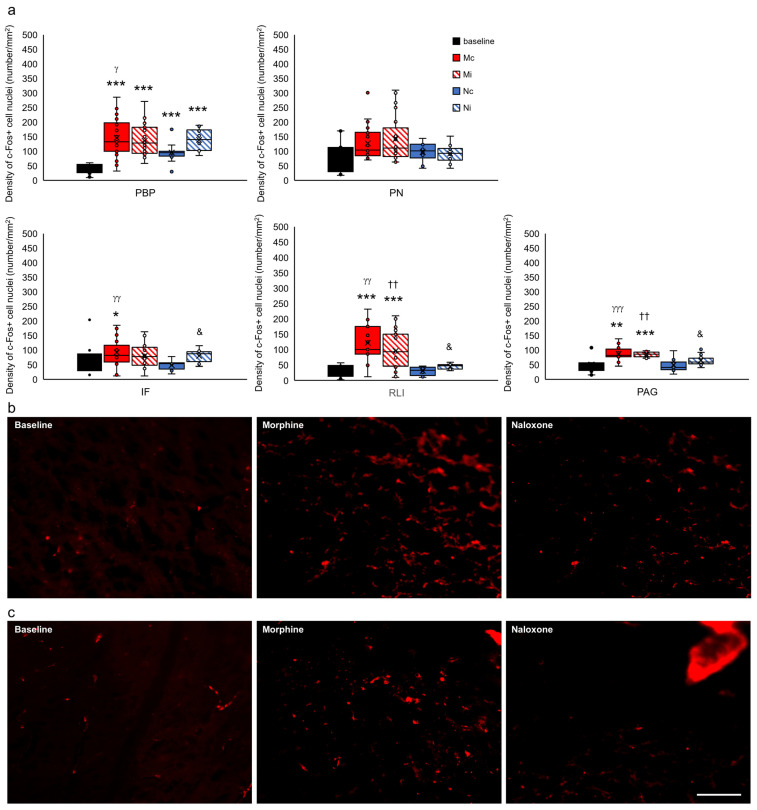
Density of c-Fos+ cell nuclei (number/1 mm^2^) in select limbic structures of the midbrain in the brains of rats that were subjected to VTA electrical stimulation (Es-VTA) only (baseline group, n = 5) (black-filled bars) and in the brains of rats administered Es-VTA and a one-time injection of morphine (1.5 µg) or naloxone (25.0 µg) injected into the PPN in the contralateral (Mc, n = 5, red-filled bars; Nc, n = 6, blue-filled bars) and ipsilateral hemispheres (Mi, n = 5, red-hatched bars; Ni, n = 6, blue-hatched bars) (**a**). Representative photomicrographs of c-Fos-labeled neurons in the PBP (**b**) and PN (**c**) structures are shown. Scale bar = 100 µm (white line: right lower corner of the last photo, panel (**c**)). Tukey’s post hoc test: * *p* < 0.05, ** *p* < 0.01, and *** *p* < 0.001 indicate a significant difference between the baseline and other experimental groups (Mc, Mi, Nc, and Ni groups); ^γ^ *p* < 0.05, ^γγ^ *p* < 0.01, ^γγγ^ *p* < 0.001 indicate a significant difference in the contralateral side groups between different injection drug: morphine or naloxone (Mc vs. Nc group); ^††^ *p* < 0.01 indicate a significant difference in the ipsilateral side groups between different injection drug: morphine or naloxone (Mi vs. Ni group); ^&^ *p* < 0.05 indicate a significant difference in the naloxone groups between different injection site: contra- or ipsilateral hemispheres (Mc vs. Mi group).

**Figure 11:**
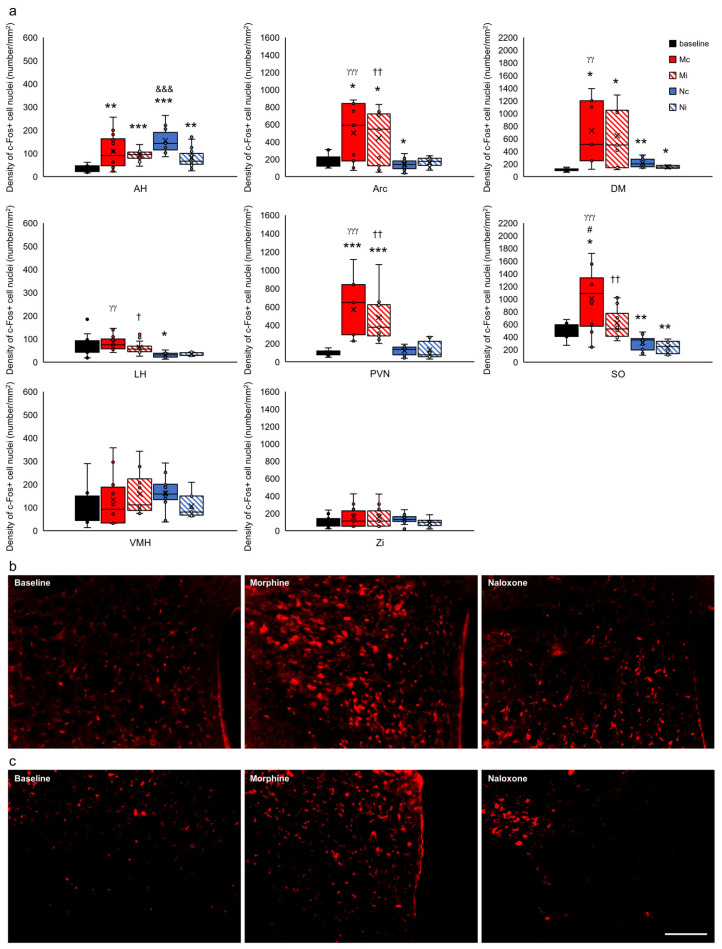
Density of c-Fos+ cell nuclei (number/1 mm^2^) in select structures of the hypothalamus and subthalamus in the brains of rats that were subjected to VTA electrical stimulation (Es-VTA) only (baseline group, n = 5) (black-filled bars) and in the brains of rats administered Es-VTA and a one-time injection of morphine (1.5 µg) or naloxone (25.0 µg) injected into the PPN in the contralateral (Mc, n = 5, red-filled bars; Nc, n = 6, blue-filled bars) and ipsilateral hemispheres (Mi, n = 5, red-hatched bars; Ni, n = 6, blue-hatched bars) (**a**). Representative photomicrographs of c-Fos-labeled neurons in the PVN (**b**) and Arc (**c**) structures are shown. Scale bar = 100 µm (white line: right lower corner of the last photo, panel (**c**)). Tukey’s post hoc test: * *p* < 0.05, ** *p* < 0.01, and *** *p* < 0.001 indicate a significant difference between the baseline and other experimental groups (Mc, Mi, Nc, and Ni groups); ^#^ *p* < 0.05 indicate a significant difference in the morphine groups between different injection site: contra- or ipsilateral hemispheres (Mc vs. Mi group); ^γγ^ *p* < 0.01, ^γγγ^ *p* < 0.001 indicate a significant difference in the contralateral side groups between different injection drug: morphine or naloxone (Mc vs. Nc group); ^†^ *p* < 0.05, ^††^ *p* < 0.01 indicate a significant difference in the ipsilateral side groups between different injection drug: morphine or naloxone (Mi vs. Ni group); ^&&&^ *p* < 0.001 indicate a significant difference in the naloxone groups between different injection site: contra- or ipsilateral hemispheres (Mc vs. Mi group).

**Figure 12:**
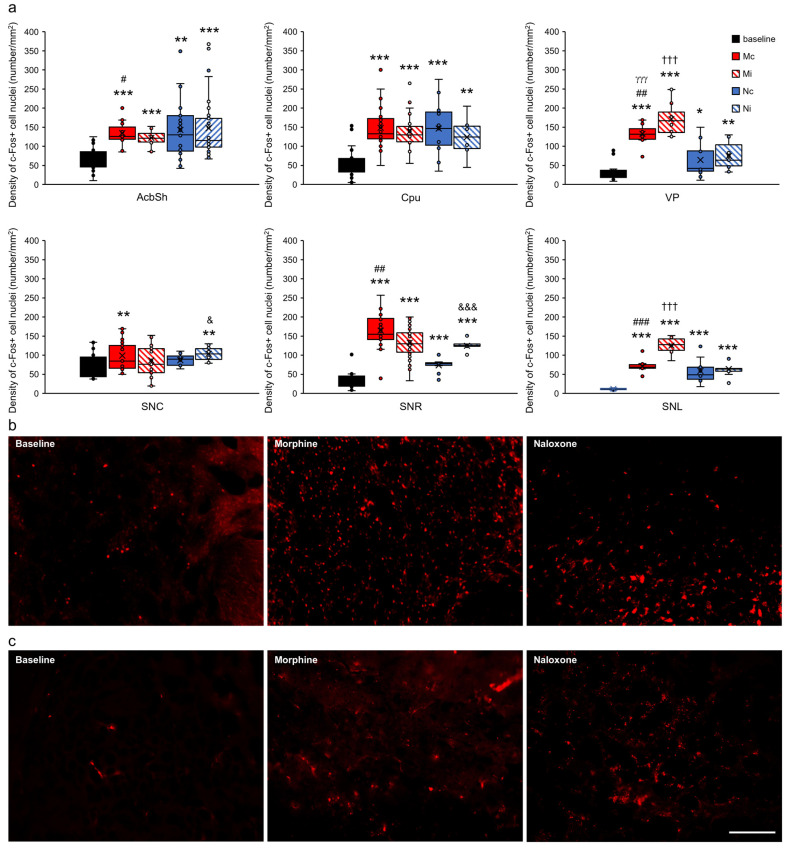
Density of c-Fos+ cell nuclei (number/1 mm^2^) in select extrapyramidal structures and ventral striatum in the brains of rats that were subjected to VTA electrical stimulation (Es-VTA) only (baseline group, n = 5) (black-filled bars) and in the brains of rats administered Es-VTA and a one-time injection of morphine (1.5 µg) or naloxone (25.0 µg) injected into the PPN in the contralateral (Mc, n = 5, red-filled bars; Nc, n = 6, blue-filled bars) and ipsilateral hemispheres (Mi, n = 5, red-hatched bars; Ni, n = 6, blue-hatched bars) (**a**). Representative photomicrographs of c-Fos-labeled neurons in the AcbSh (**b**) and Cpu (**c**) structures are shown. Scale bar = 100 µm (white line: right lower corner of the last photo, panel (**c**)). Tukey’s post hoc test: * *p* < 0.05, ** *p* < 0.01, and *** *p* < 0.001 indicate a significant difference between the baseline and other experimental groups (Mc, Mi, Nc, and Ni groups); ^#^ *p* < 0.05, ^##^ *p* < 0.01, ^###^ *p* < 0.001 indicate a significant difference in the morphine groups between different injection site: contra- or ipsilateral hemispheres (Mc vs. Mi group); ^γγγ^ *p* < 0.001 indicate a significant difference in the contralateral side groups between different injection drug: morphine or naloxone (Mc vs. Nc group); ^†††^ *p* < 0.001 indicate a significant difference in the ipsilateral side groups between different injection drug: morphine or naloxone (Mi vs. Ni group); ^&^ *p* < 0.05, ^&&&^ *p* < 0.001 indicate a significant difference in the naloxone groups between different injection site: contra- or ipsilateral hemispheres (Mc vs. Mi group).

For clarification of presentation, 35 studied brain structures were grouped into functional units for comparisons of the control group with all of the experimental groups and presented as the limbic cortex (Figure 7), select structures of the septal system (Figure 8), limbic nuclei of the thalamus (Figure 9) and midbrain (Figure 10), hypothalamus and subthalamus (Figure 11), and the extrapyramidal structures with the ventral striatum (Figure 12).

In select structures of the limbic cortex, such as the cingulate cortex, areas of Cg1 and Cg2, and retrosplenial granular (RSG) and agranular cortex (RSA), statistical analysis of c-Fos density did not appear to be affected by the type of injected drug (morphine, naloxone, and baseline) and the hemisphere of injection (ipsi-/contralateral hemisphere) (Cg1, F_(2,83)_ = 2.197, *p* > 0.05; Cg2, F_(2,105)_ = 2.434, *p* > 0.05; RSG, F_(2,45)_ = 0.406, *p* > 0.05; and RSA, F_(2,44)_ = 0.685 *p* > 0.05). However, there was a relationship between c-Fos density in these structures and the type of injected substance (Cg1, F_(2,83)_ = 28.631, *p* < 0.001; Cg2, F_(2,105)_ = 124.267, *p* < 0.001; RSG, F_(2,45)_ = 147.806, *p* < 0.05; and RSA, F_(2,44)_ = 5.081, *p* < 0.01). Thus, after morphine injection, an increase of c-Fos compared to the baseline in Cg1, Cg2, RSG, and RSA (rats with morphine injection vs. baseline, *p* < 0.001) was found. This increase was also observed compared to morphine and naloxone injection (rats with morphine injection vs. rats with naloxone injection (Cg1, *p* < 0.001; Cg2, *p* < 0.001; and RSG, *p* < 0.001). Post hoc test analysis of the experimental groups, divided into drug type and hemisphere of injection, showed that morphine injection into the PPN, both in the contralateral (Mc group: red-filled bars) and ipsilateral hemispheres (Mi group: red-hatched bars), increased c-Fos expression in comparison to the baseline (baseline group: black-filled bars) in Cg1, Cg2, RSG (*p* < 0.001), and RSA (only in the ipsilateral hemisphere) (*p* < 0.05) (Figure 7a). In the case of naloxone injection, the higher density of nuclei with c-Fos protein was also observed in Cg1 in the Ni group (*p* < 0.01) (Figure 7a; Ni group: blue-hatched bars) and in Cg2 in the Nc group (*p* < 0.001) (Figure 7a; Ni group: blue-filled bars).

The analysis of the c-Fos+ density in the select structures of the septal showed a relationship between the type of drug and hemisphere only in the intermediate part of the lateral septal nucleus (LSI) (F_(2,52)_ = 4.647, *p* < 0.05), medial septal nucleus (MS) (F_(2,66)_ = 40.836, *p* < 0.001), and nucleus of the vertical limb of the diagonal band (VDB) (F_(2,54)_ = 7.643, *p* < 0.001) as compared to the dorsal (LSD) (F_(2,82)_ = 2.502, *p* > 0.05) and ventral part of the lateral septal nucleus (LSV) (F_(2,34)_ = 0.581, *p* > 0.05) and nucleus of the horizontal limb of the diagonal band (HDB) (F_(2,38)_ = 1.971, *p* > 0.05). In addition, a relationship between c-Fos+ density and the type of injection substance in all septal structures was found (LSD, F_(2,82)_ = 318.435, *p* < 0.001; LSI, F_(2,52)_ = 62.069, *p* < 0.001; LSV, F_(2,34)_ = 127.590, *p* < 0.001; MS, F_(2,66)_ = 74.502, *p* < 0.001; VDB, F_(2,54)_ = 123.446, *p* < 0.001; and HDB, F_(2,38)_ = 46.739, *p* < 0.001). Tukey’s post hoc test showed that more c-Fos+ density was observed in all septal structures in the Mc (red-filled bars) and Mi groups (red-hatched bars) (*p* < 0.01 and *p* < 0.001) in comparison to the baseline (black-filled bars) (Figure 8a–c). In the Nc and Ni groups, more c-Fos was only observed in LSD (Nc and Ni: *p* < 0.001), LSI (Nc, *p* < 0.01 and Ni, *p* < 0.001), and HDB (Nc, *p* < 0.01 and Ni, *p* < 0.001) (Figure 8a; blue-filled and blue-hatched bars).

In the thalamic limbic nuclei, analysis of c-Fos protein showed a relationship between injection and hemisphere only in the anterodorsal thalamic nucleus (AD) (F_(2,33)_ = 6.844, *p* < 0.01). Moreover, the density of c-Fos+ in this structure varied depending on the injection agent (F_(2,33)_ = 746.084, *p* < 0.001), with more c-Fos+ found in rats after injection of morphine (Mc and Mi groups) (Figure 9a, red-filled and hatched bars) or naloxone (Nc and Ni groups) (Figure 9a, blue-filled and blue-hatched bars) compared to rats without injection (baseline group) (*p* < 0.001) (Figure 9a, black-filled bars). A similar effect was observed in the analysis of the distribution of c-Fos within the hemisphere, where more c-Fos+ nuclei were observed in the contralateral hemisphere (F_(1,33)_ = 18.276, *p* < 0.001). Post hoc test showed this relationship only in rats injected with morphine (Mc vs. Mi, *p* < 0.001) (red-filled and red-hatched bars) (Figure 9a). Analysis of subsequent limbic structures of the thalamus showed only the dependence of c-Fos+ density from the type of injected agent (rats with morphine/naloxone/without injection). These structures were anteromedial (AM) (F_(2,33)_ = 41.620, *p* < 0.001) and anteroventral (AV) (F_(2,50)_ = 29.242, *p* < 0.001) and mediodorsal thalamic nucleus (MD) (F_(2,58)_ = 39.812, *p* < 0.001), lateral (LHb) (F_(2,53)_ = 37.417, *p* < 0.001), and medial habenular nucleus (MHb) (F_(2,53)_ = 98.866, *p* < 0.001), where more c-Fos+ was observed in AM and AV in rats with Mc and Mi groups (red-filled and red-hatched bars) and in rats with Nc and Ni groups (blue-filled and blue-hatched bars), next in MHb and LHb in rats with Mc and Mi groups (red-filled and red-hatched bars), and also in MD in rats with Nc group (blue-filled bars) as compared to the baseline group (black-filled bars) (Figure 9a–c).

In the limbic midbrain structures, only drug-dependent changes of c-Fos density in the parabrachial pigmented nucleus (PBP) (F_(2,125)_ = 61.077, *p* < 0.001), periaqueductal gray (PAG) (F_(2,85)_ = 20.416, *p* < 0.001), rostral linear nucleus (RLi) (F_(2,110)_ = 37.428, *p* < 0.001), and interfascicular nucleus (IF) (F_(2,104)_ = 3.211, *p* < 0.05) were shown. The analysis of the individual effects showed that the increased c-Fos+ nuclei were in rats injected with morphine in the structures of PBP, RLi, PAG (Mc and Mi groups) (*p* < 0.001 and *p* < 0.01) (red-filled and red-hatched bars), and IF (only Mc group) (*p* < 0.05) (red-filled bar) (Figure 10a–c) as compared to rats without any injection included in the baseline group (black-filled bars) (Figure 10a–c). In turn, in rats injected with naloxone (Nc and Ni groups), an increase in c-Fos+ nuclei was observed only in PBP (*p* < 0.001) (blue-filled and blue-hatched bars) compared to rats that did not receive naloxone injection (baseline group) (black-filled bar) (Figure 10a).

In the hypothalamus, an effect of the drug and injection hemisphere on c-Fos density in the anterior hypothalamic area (AH) (F_(2,71)_ = 4.967, *p* < 0.01) and supraoptic nucleus of the hypothalamus (SO) (F_(2,62)_ = 3.132, *p* < 0.05) was found. Furthermore, in these structures, dependence of c-Fos+ on the injection hemisphere was observed (AH, F_(1,71)_ = 8.800, *p* < 0.01 and SO, F_(1,62)_ = 4.971, *p* < 0.05). In addition, in these structures (AH and SO) and other hypothalamic structures such as the arcuate nucleus (Arc), dorsomedial nucleus (DM), lateral (LH), and paraventricular nucleus (PVN), a higher density of nuclei with c-Fos+ protein was observed depending on the injected agent (AH, F_(2,72)_ = 23.349, *p* < 0.001; SO, F_(2,62)_ = 25.167, *p* < 0.001; Arc, F_(2,79)_ = 25.398, *p* < 0.001; DM, F_(2,41)_ = 19.213, *p* < 0.001; LH, F_(2,54)_ = 6.313, *p* < 0.01; and PVN, F_(2,46)_ = 29.732, *p* < 0.001). Detailed post hoc analysis showed a significant increase in c-Fos+ in rats injected with morphine (red-filled and red-hatched bars) and naloxone (blue-filled and blue-hatched bars) compared to rats that did not receive the drug (black-filled bars) in AH (Mc, Mi vs. baseline, *p* < 0.01 and *p* < 0.001) (Nc, Ni vs. baseline, *p* < 0.001 and *p* < 0.01), Arc (Mc, Mi vs. baseline, *p* < 0.05) (Nc vs. baseline, *p* < 0.05), DM (Mc, Mi vs. baseline, *p* < 0.05) (Nc, Ni vs. baseline, *p* < 0.01 and *p* < 0.05), LH (Nc vs. baseline, *p* < 0.05), PVN (Mc, Mi vs. baseline, *p* < 0.001), and SO (Mc vs. baseline, *p* < 0.05) (Nc, Ni vs. baseline, *p* < 0.01) (Figure 11a–c). There was no change in c-Fos density in the ventromedial hypothalamic nucleus (VMH) (drug x hemisphere, F_(2,49)_ = 1.066, *p* > 0.05) and zona incerta (ZI) (drug x hemisphere, F_(2,62)_ = 0.342, *p* > 0.05) (Figure 11a). 

The influence of agent and injection hemisphere on c-Fos density in the extrapyramidal structures mainly affected the reticular (SNR) (F_(2,142)_ = 11.688, *p* < 0.001) and lateral parts of the substantia nigra (SNL) (F_(2,52)_ = 12.797, *p* < 0.001). Furthermore, an increase in the density of nuclei with c-Fos protein depended on the type of injected drug in all analyzed structures such as the Acb, part shell (Acbsh) (F_(2,165)_ = 35.827, *p* < 0.001), caudate putamen (Cpu) (F_(2,93)_ = 29.218, *p* < 0.001), substantia nigra, compact part (SNC) (F_(2,154)_ = 10.726, *p* < 0.001), SNR (F_(2,149)_ = 200.939, *p* < 0.001), SNL (F_(2,52)_ = 102.632, *p* < 0.001), and ventral pallidum (VP) (F_(2,82)_ = 109.850, *p* < 0.001). The increase in c-Fos protein density, which depended on the injection hemisphere (ipsi-/contralateral hemispheres), was observed only in SNL (F_(1,52)_ = 16.322, *p* < 0.001) and in VP (F_(1,82)_ = 5.218, *p* < 0.05). In the remaining analyzed structures, this relationship was not found (AcbSh, F_(1,165)_ = 0.062, *p* > 0.05; Cpu, F_(1, 93)_ = 0.838, *p* > 0.05; SNC, F_(1,154)_ = 0.782, *p* > 0.05; and SNR, F_(1,149)_ = 1.254, *p* > 0.05). 

This was confirmed by a post hoc analysis of comparisons within individual experimental groups, taking into account their hemispheric differentiation (Mc, Mi, Nc, and Ni vs. baseline) (Figure 12a–c).

### 2.3. Histological Verification

**Figure 13:**
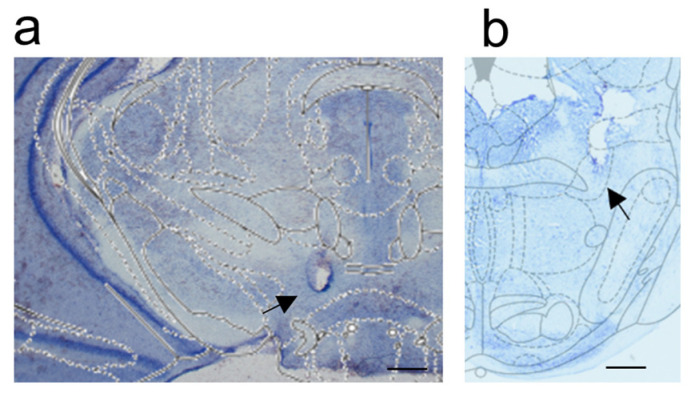
Photographs of selected brain sections from representative rats in the Mc-5 group stained with Nissl staining with localization of the electrode in the VTA (distance from bregma −4.80 mm; black arrow—the position of the electrode tip) (**a**) and the cannula in the PPN (distance from bregma −8.00 mm; black arrow—the position of the cannula tip) (**b**) with templates taken from the rat brain atlas [28] (a magnifier Stemi 508; Zeiss with camera Axiocam 105 color; Zeiss and with integrated software Zen Digital Imaging (version 3.2 (blue edition); Zeiss, magnification 2.5 × 10, scale bar 1 mm—right lower corner of the photos).

The localization of both the electrode and the cannula was confirmed by histology for all experimental groups in the behavioral studies and the baseline group used for immunofluorescence studies (Nissl staining) (Figure 13). In all of the experimental groups in which rats received morphine injection (Mc-1, Mc-2, Mc-3, Mc-4, Mc-5 and Mi-1, Mi-2, Mi-3, Mi-4, Mi-5) (n = 50) or naloxone injection into the PPN (Nc-1, Nc-2, Nc-3, Nc-4, Nc-5 and Ni-1, Ni-2, Ni-3, Ni-4, Ni-5 groups) (n = 36), and a group of rats with only Es-VTA without injection into the PPN (baseline group) (n = 5), the electrodes were localized to the rostral VTA (from −4.80 mm to −5.30 mm posterior to bregma) (Figure 13a, Table 1). The cannula tips were confirmed in the central and caudal parts of the PPN (from 7.64 mm to 8.00 mm posterior to bregma) in rats in all of the experimental groups (rats after morphine injection: Mc-1, Mc-2, Mc-3, Mc-4, Mc-5 and Mi-1, Mi-2, Mi-3, Mi-4, Mi-5 groups and rats after naloxone injection: Nc-1, Nc-2, Nc-3, Nc-4, Nc-5 and Ni-1, Ni-2, Ni-3, Ni-4, Ni-5 groups) according to the assigned templates from the rat brain atlas [28] with respect to the distance from the bregma point (in the upper and lower parts of the image) during the image captures (Figure 13b, Table 1).

## 3. Discussion

This study examined the influence of OR activation in the PPN on FR induced by Es-VTA. It was shown that the excitation of OR by morphine and their blocking by naloxone within the PPN nucleus caused a change in motivational behavior as well as a change in neuronal activation, where OR into the PPN resulted in marked, dose-dependent changes in FR latency. Morphine doses of 1.25 and 1.5 µg significantly reduced the FR induced by unilateral Es-VTA, whereas morphine doses of 0.25 and 0.5 µg did not affect this behavior. The opposite effect was observed after naloxone injection, where the lowest doses of 2.5 and 5.0 μg shortened the FR latency. Still, the highest naloxone dose of 25.0 μg did not cause changes in this reaction. These results corresponded with increased neuronal activity, measured by c-Fos protein expression, in most of the analyzed brain structures, especially after injection of morphine (in both the Mc and Mi groups). Moreover, the behavioral analysis did not find differences in the FR latency and neuronal activation between the groups that received the opioid injections in the ipsi- or contralateral hemisphere (Mc vs. Mi and Nc vs. Ni). 

So far, it is known that psychotropic substances directly or indirectly increase DA levels in Acb through specific interactions with various brain regions, and the somatodendritic ML regions (VTA region) together with their neuronal terminations (Acb nucleus) appear to be crucial for the regulation of the reward system [29]. Both morphine, which is an opioid agonist, and naloxone, which is an opioid antagonist, affect the µ receptors in the PPN [30,31,32]. Our research showed that during FR induced by Es-VTA, a change of neuronal activity in the PPN nucleus occurred immediately after the temporary activation/inactivation of μ receptors within this nucleus. Based on previous studies with intra-cerebral injections of morphine, five different injection doses were used in these experiments: 0.25 µg/0.5 µL [33,34], 0.5 µg/0.5 µL [34,35], 1.0 µg/0.5 µL [36], 1.25 µg/0.5 µL, and 1.5 µg/0.5 µL [36,37]. For naloxone, three different injection doses were used: 2.5 µg/0.5 µL and 25 µg/0.5 µL [32], and 5 µg/0.5 µL [37].

The VTA is a neuronal structure involved in modulating psychostimulant abuse, including opioids, and its output is essential for the rewarding effects of addictive drugs [38,39,40,41]. Apart from motor control, the PPN is also involved in reward processing, where its neuronal activity may change in response to reward signals [42], and pharmacological inhibition of these neurons may alter established addiction behavior [43]. Optogenetic and chemogenetic research provides evidence for the heterogeneity of the PPN in terms of anatomy and its connectivity with many structures, including parts of the basal ganglia, subthalamic nucleus (STN), SN, and VTA [44]. Cholinergic, glutamatergic, and GABAergic neurons of the PPN form concurrent connections onto midbrain A9–A10 dopaminergic neurons and other neurons in the SN and VTA structures [45,46]. It has been found that cholinergic neurons represent a minority of all neurons in the PPN and are found intermingled with a large number of GABAergic and glutamatergic neurons [26,47]. The distribution of cholinergic neurons within the PPN varies along the rostrocaudal axis of this nucleus. There are a few cholinergic neurons in the rostral part of the PPN. Still, in the caudal part of the PPN, this number is significant [48]. In the rostral part of the PPN, cholinergic neurons primarily innervate motor structures such as the SNC and dorsolateral striatum, whereas, within the caudal part of the PPN, these neurons innervate the limbic structures such as the VTA and dorsomedial striatum [24,44,48,49]. In addition, it was found that the PPN sends glutamatergic projections to the SNC and VTA. On the other hand, it was found too that there is a link between cholinergic activation and OR in the PPN and DA activity of the VTA [50]. The effect of the injection of DAMGO (a selective μ-OR agonist) in the PPN depended on the injection site [51]. The reduced DAMGO effect was related to the increased distance of the injection site from the population of cholinergic neurons in its nucleus. Thus, our research aimed to investigate changes in the latency of ML stimulation-induced FR and the role of the PPN opioid system (especially in its caudal part) in this behavior via alterations in the OR activation in the PPN.

The process of PPN activation is embroiled with the animal’s intentional search for psychostimulants [41]. Injections of µ agonist into the PPN are DA-dependent and involve variable stimulation of VTA DA neurons [52]. In our experiments, the injection of morphine in small doses (0.25 μg/0.5 μL, 0.5 μg/0.5 μL, and 1 μg/0.5 μL) did not significantly change the FR latency compared to the control (ACSF injection). At higher morphine doses (1.25 μg/0.5 μL and 1.5 μg/0.5 μL), most animals experienced significant impairment in the FR latency, which was directly proportional to the increased dose of morphine. The opposite effect was observed after injection of naloxone into the PPN nucleus, where it turned out that the blockade of µ-receptors in the PPN correlated with a shorter FR latency compared to the control (ACSF injection), and similar to the morphine injections, was dose-dependent. A shortening of the FR latency at the lowest dose of naloxone (2.5 µg) and no change of the FR latency at the highest administered dose (25 µg) was observed.

The µ-opioid system could arouse higher-level cognitive function through modulation of valuation, motivation, and control circuits dense in µ-OR, which included the orbitofrontal cortex, basal ganglia, Amg, anterior Cg1 and Cg2, and PFC [53]. The process of decision making and cognitive control is altered by opioids by increasing the subjective value of reward and reducing the aversive arousal [53,54]. Essential for the interpretation of the results of our study is the fact that the motivational effects induced by food and morphine are isometric, and therefore, morphine “replaces food” [55]. These authors classified the reward resulting from morphine and food to be at the same level and that morphine overshadows the reward effect of food consumption. Moreover, it turned out that the induced PPN lesions did not affect the preferences of the environment (an environment with the possibility of morphine self-administration compared to an environment without the possibility of morphine self-administration). The results from our study and the aforementioned studies may suggest that the prolongation of the latency of the reaction, and thus the motivation, was the receipt of the reward in the form of morphine. Rats, like humans, are driven by the prospect of reward. Meeting basic needs is rewarded by the release of the DA reward system at neural terminals. Animals may become unmotivated to eat because they have already been rewarded [55]. The dose-dependence effect that we observed (deterioration of the FR latency related to the increase in the intra-cerebral dose of morphine) seems to confirm this theory. Moreover, the behavioral effect was more noticeable when both morphine and naloxone injections into the PPN nucleus were applied to the ipsilateral hemisphere than when injected into the contralateral hemisphere compared to the ACSF injection. However, no interhemispheric differentiation was found within the experimental groups (Mc vs. Mi and Nc vs. Ni). It is known that the rostral part of the PPN innervates the SNC predominantly, but the caudal part of the PPN innervates both the SNC and the VTA [56,57]. Furthermore, these projections are largely unilateral [58]. However, analysis of the separate neuronal connections of the PPN and VTA showed that the number of cholinergic PPN–VTA connections coincided in both hemispheres [25]. In contrast, the non-cholinergic PPN to VTA connections are more abundant in the ipsilateral than the contralateral hemisphere [29]. Thus, our result may indicate that the connections between the PPN and VTA are slightly more strongly pronounced in the ipsilateral hemisphere. This result (especially evident in behavioral tests) may also relate to the hemispheric effect (ipsi-/contralateral hemisphere) and the injection site. Where we aimed at the caudal part of the PPN apart from cholinergic neurons, there is also a population of glutamatergic neurons. 

Opiates appear to be involved in the rewarding effects of food [59]. In our study, besides behavioral experiments, we compared the activity of c-Fos nuclei in select structures, including the limbic cortex, septum, thalamus, hypothalamus, and extrapyramidal and ventral striatum structures and limbic structures of the midbrain, in rats subjected to Es-VTA with morphine or naloxone injections in the PPN versus rats that only received Es-VTA. In most of the analyzed structures, an increase in the density of c-Fos+ nuclei was observed in rats administered morphine into the PPN. On the other hand, after injection of naloxone into the PPN nucleus, no increase in the density of c-Fos+ nuclei was observed in most structures of the limbic cortex (except for Cg1 in Ni group), septum (except for LSD, LSI, and HDB in both Nc and Ni groups), hypothalamus (except AH, DM, and SO nuclei in both Nc and Ni groups), and midbrain (except PBP in both Nc and Ni groups). The exception was the limbic nuclei of the thalamus (including AD, AM, AV, and MD) and all nuclei classified as extrapyramidal and ventral striatum (AcbSh, Cpu, SNR, SNL, and VP), where an increase in the density of the c-Fos protein was observed after injection of naloxone into both the ipsi- and contralateral hemispheres. However, the comparative increase in c-Fos+ nuclei density in these structures after naloxone injection was not as high as after morphine injection, which was given to both groups of rats with injections into the ipsi- and contralateral hemispheres. 

So far in the literature, connections between the PPN and VTA have been shown to be both cholinergic and glutamatergic [48,60]. PPN projections that are not cholinergic also have a strong influence on the activity of DA cells in the VTA and the formation of motivation [46,61,62]. Thus, these projections contribute directly or indirectly to changes in DA neuronal activity and plastic changes in the VTA, which have been linked to the process of addiction development [63]. In turn, cholinergic neurons of the PPN provide a reinforcing signal to DA neurons. Still, these neurons only constitute a fraction of the PPN in contrast to glutamatergic neurons in the PPN, which also modulate the activity of DA neurons [24]. Cholinergic efferents from the PPN to the VTA are a part of a circuit that includes the medial PFC [41,64]. The PFC has glutamatergic connections with the VTA. In addition, the VTA sends dopaminergic and GABAergic efferents to the Acb. Stimulation of excitatory inputs from the PPN increases the burst firing of DA neurons in the VTA, which could suggest that the PPN is partially responsible for the regulation of the reward and motivational functions of the VTA [41,45,65]. PPN cholinergic neurons projecting to the VTA and ventral substantia nigra pars compacta (vSNc) are intermingled, and some of these neurons project to both the VTA and vSNc besides separate, parallel projections to these structures [46]. Therefore, the release of mesolimbic DA is regulated by a number of factors [65]. 

VTA participation in the morphine reward is consistent with two parallel glutamate and/or GABA reward output pathways from the PPN [66]. It was found a large amount of acetylcholine reactivity in the VTA and SN. This cholinergic innervation came mainly from the PPN [67]. Thus, acetylcholine in the VTA and SNC causes an increase in the frequency of DA and non-DA cell discharge, which leads to increased DA levels selectively in the Acb and thus influences an increase in locomotor activity [68], and is implicated in the rewarding effects of food [69]. Thus, cholinergic inputs from the PPN to the VTA are also significant because lesions of the PPN strongly attenuate morphine-induced forebrain DA elevations [70]. It is known that in the PPN, there are also µ-OR [51,71] and, as in other brain structures, they can be found on cholinergic cells [72] and GABAergic axon terminals [73]. It is also known that activation of the OR in the PPN causes inhibition of the release of these neurotransmitters from this nucleus [37,74], especially PPN cholinergic inputs from its caudal part to the VTA. This information is then processed in the Acb and other reward regions, including the LH, VP, and PFC [75,76].

In our behavioral studies, we observed that injection of morphine into the caudal part of the PPN increased the FR latency induced by Es-VTA. On the other hand, the FR latency was reduced after injection of naloxone into the caudal part of this nucleus. In addition, the behavioral effect after morphine and naloxone administration was dose dependent. Interestingly, c-Fos+ nucleus density in the VTA, within the PN (target site of stimulation), was the same in both experimental groups as in the control group. Changes were also not observed in the SNC in the group of rats administered morphine into the PPN in the ipsilateral hemisphere. A similar result was observed in the LH, which is directly connected and adjacent to the VTA. The stimulation of this structure also induces both food and reward reactions [5]. After injection of morphine into the PPN and Es-VTA, the density of c-Fos nuclei did not change in the LH. After injection of naloxone into the PPN and Es-VTA, the density of c-Fos nuclei was considerably lower in the LH. An interesting result also concerned the LHb. It is known that the LHb is involved in negative reward processes, i.e., processes that encode situations, where no reward is available [77]. The VTA sends GABAergic projection to the LHb [78]. In turn, the LHb sends modest glutamatergic projection to the VTA DA neurons and PFC glutamatergic neurons [79,80], and robust excitatory projection to GABAergic neurons in the rostromedial tegmental nucleus (RMTg) and posterior VTA [78,81]. Therefore, the LHb electrical stimulation inhibits the firing of up to 97% of the midbrain dopaminergic neurons [82]. This study showed more activation of c-Fos nuclei in the LHb in the case of injection of morphine into the PPN. On the other hand, after the injection of naloxone at its highest dose, the density of c-Fos in the LHb did not change compared to the control. Thus, the injection of morphine into the PPN weakened the reward, especially at its highest doses. The consequence was increased LHb activation, but at the highest dose of naloxone, this effect was the opposite. Thus, perhaps after morphine injection into the caudal part of the PPN, the observed increase in FR latency induced by Es-VTA could be caused by the temporary disappearance from cholinergic excitation in the caudal part of the PPN only for DA neurons in the VTA. Thus, this may result from reward devaluation, which signals and directs rats that previous more gratifying circumstances are no longer available to them. In addition, this result suggests numerous heterogeneous connections between the PPN and VTA, and the VTA has many DA and non-DA brain structures that control feeding in different ways, which are not fully known yet. 

In conclusion, our results show that the specified modulation in OR activity can support or impair motivational and behavioral effects within the PPN nucleus. Moreover, the results indicate that the mechanisms of VTA neuronal activity, its projections, and the PPN–VTA interdependence are not fully understood.

## 4. Materials and Methods

The rats (Tri-City Central Animal Laboratory, Research and Service Center of the Medical University of Gdansk, Poland) (male Wistar; n = 91), weighing 200–300 g, were subjected to taming procedures and Es-VTA along with intra-cerebral injection into the PPN. These procedures were carried out with due care and attention to the comfort of the animals to minimize stress on them. Previous studies have described these procedures [4,27].

### 4.1. Behavioral Procedures

The surgery was performed using 1–2.5% isoflurane (Aeranne; Baxter, IL, USA) anesthesia. Ninety-one rats were used in the behavioral study. All rats underwent bilateral implantation of VTA stimulation electrodes and unilateral implantation of injection cannulas into the PPN nucleus and a reference electrode on the surface of the skull. Methods of stimulation and reference electrodes preparation have been previously described [4,83]. 

For this experiment, the monopolar electrodes for VTA were handmade of stainless-steel wire with a diameter of 0.127 mm and insulated with epoxy varnish along the entire length, except for the flat cut end (type 008SW, Bilaney Consultants GmbH, Düsseldorf, Germany). The wire was soldered to a gold-plated connector (H2 plug contact No. 74-7004-02, Unitra Eltra Radio Factory, Bydgoszcz, Poland) to enable the connection with its counterpart soldered to the stimulator cable (G2 socket contact No. 74-7005-02, Unitra Eltra Radio Factory, Bydgoszcz, Poland). The reference electrode was an analogous gold-plated joint connected with a jewelry screw. Guide (type C312G) and internal cannulas (type C313I) were used (Bilaney Consultants GmbH). 

The coordinates for implantation were: VTA: 4.80–5.20 mm posterior to the bregma, 1.00 mm lateral to the midline, and 8.00–8.10 mm ventral to the skull surface (skull leveled) and PPN: 7.80–8.00 mm posterior to the bregma, 1.70 mm lateral to the midline, and 7.00–7.20 mm ventral to the skull surface (skull leveled). Stimulation and injection procedures started after a 7-day recovery from the surgery.

#### 4.1.1. Experimental Groups

The method for assessing the FR of the animals during Es-VTA has been previously described [4]. In this study, the procedure involved induction of FR by Es-VTA and subsequent injection of morphine (Morphine hydrochloride, Tocris Bioscience, Bristol, UK) or naloxone (Naloxone hydrochloride dihydrate, Sigma-Aldrich, St. Louis, MO, USA) into the contralateral or ipsilateral hemisphere of the PPN. The behavioral study was divided into two main tests: (1) the effect of morphine microinjection in the PPN on FR induced by Es-VTA (n = 50) and (2) the effect of naloxone microinjection in the PPN on FR induced by Es-VTA (n = 36). In addition, the animals were also divided into different experimental groups depending on the Es-VTA and PPN-injection hemispheres and injection dose in each of the tests. Moreover, each animal (except injection with artificial cerebrospinal fluid (ACSF) as a control) (ACSF, Tocris Bioscience, Bristol, UK) received only one injection of a specified drug (morphine or naloxone) at only one dose. Thus, in the case of morphine, there were 10 groups (n = 5 in each group) based on 5 different doses of morphine (0.25, 0.5, 1.0, 1.25, and 1.5 µg) and injection sites (contralateral or ipsilateral to the Es-VTA). The groups of animals were as follows: Mc-1, Mc-2, Mc-3, Mc-4, Mc5 (n = 25) and Mi-1, Mi-2, Mi-3, Mi-4, Mi-5 (n = 25). A similar procedure for dividing the animals into experimental groups was performed for the naloxone injections in the PPN (n = 36). Thus, based on three different doses of naloxone (2.5, 5.0, 25.0 µg) and the PPN areas they were injected in (contralateral or ipsilateral hemisphere to the Es-VTA), 6 experimental groups were created (in each group, n = 6): Nc-1, Nc-2, Nc-3 (n = 18) and Ni-1, Ni-2, Ni-3 (n = 18). Therefore, animals that received injections of 5 different doses of morphine and animals that received 3 different doses of naloxone injected in the contralateral and ipsilateral hemispheres belonged to separate experimental groups described above (injection with morphine: 10 experimental groups and injection with naloxone: 6 experimental groups). The FR in each group was assessed quantitatively based on (1) the threshold frequency and (2) the latency/frequency curve, according to a detailed description by a previous study [4].

Es-VTA sessions were performed on the same day as the injection (3 min after injection).

#### 4.1.2. Electrical Stimulation

After a recovery period of 7 days from implantation, the rats were screened for Es-VTA-induced FR. The rats had free access to food (standard compound food for laboratory animals, Labofeed; Miaskowo, Poland) and water during the entire experiment. 

The Es-VTA procedure consisted of a screening and a proper procedure. Es-VTA was performed with a stimulator (Hugo Sachs Elektronik Type 215), where pulse duration, pulse frequency, and stimulation intensity were monitored with an oscilloscope (GOS-620 GW Instek oscilloscope, TME Electronic Components). Current parameters for Es-VTA in the screening procedure: 50 Hz (frequency) and 0.1 ms (pulse width), whereas the intensity of current (90–280 µA) was adjusted individually for each animal. Each Es-VTA consisted of 30 s trials (20 s rest between these trials). As soon as the current was determined, the FR induced by Es-VTA was established so that the latency of the response was within 5 s. After fixation of the latency in the FR induced by Es-VTA, the appropriate experimental procedure (i.e., proper Es-VTA) was performed. In the proper Es-VTA procedure, an electric current of constant width and intensity was used (determined individually for each animal during the screening Es-VTA procedure) but at a variable frequency. The frequency varied from trial to trial (ranging between 17.71 and 81.38 Hz with a between-trial increment in stimulation frequency of 10% of the previous value). There were 17 trials in a trial block. Four blocks of trials were performed each day. 

The results were analyzed after each Es-VTA day, and the threshold for FR induced by Es-VTA was determined. The FR threshold was defined as each rat’s stimulation frequency at which FR latency was 20 s (linear interpolation method for FR latency/current frequency function). When the FR threshold was stable (i.e., it did not change by more than 10% in 3 consecutive test days), the rats were subjected to intra-PPN injections.

#### 4.1.3. Intra-Cerebral Microinjection

After the Es-VTA screening procedure at which the threshold had stabilized, all rats received a control injection of ACSF to control for possible effects of the solvent and/or mechanical irritation of the PPN tissue. Immediately after injection, the stability of the FR threshold was checked in all the experimental groups. In all of the animals tested, no changes in the FR threshold were found both before and after ACSF injection into the PPN. The next step was a single injection with a specific drug. Thus, all rats from the Mc-1, Mc-2, Mc-3, Mc-4, Mc-5 and Mi-1, Mi-2, Mi-3, Mi-4, Mi-5 groups (n = 50) received an injection of morphine, a specific agonist of the OR, only once. The injection consisted of only 1 out of 5 doses of morphine: 0.25, 0.5, 1.0, 1.25, or 1.5 µg. All rats from the Nc-1, Nc-2, Nc-3 and Ni-1, Ni-2, Ni-3 groups (n = 36) received one injection of naloxone, an antagonist of the OR, in a dose of 2.5, 5.0, or 25.0 µg. Both agents were dissolved in 0.5 µL ACSF. The injections were either ipsilateral or contralateral position to the Es-VTA performed through the chronically implanted guide cannulas (type C312G; Bilaney Consultants GmbH, Düsseldorf, Germany) and using a 10 µL Hamilton syringe (0.28 mm diameter) with the pump (Kd Scientific; RoHS Compliant, Holliston, MA, USA) (0.5 µL injection volume). The internal cannula was then replaced with an obturator, and 2–3 min after injection, rats were then tested for FR induced by Es-VTA. 

#### 4.1.4. Histology Staining

All animals were killed two hours after the end of Es-VTA (pentobarbital, 0.9% saline, phosphate-buffered 4% formaldehyde). 

To determine the placement of the electrode in the VTA and cannula in the PPN, Nissl staining in select sections was performed from 4.52 to 5.60 mm and from 7.30 to 8.30 mm sections (the thickness of each section was 20 µm). The sections were mounted on gelatin-coated slides, stained with Cresyl violet perchlorate (Sigma-Aldrich, St. Louis, MO, USA), and dehydrated, and coverslips were placed on top using DPX for histology (Sigma-Aldrich, St. Louis, MO, USA). Micro photos of the sections were taken with simultaneous application of templates derived from the brain atlas of rats [28] using a magnifier (Stemi 508; Zeiss, Oberkochen, Germany) and camera (Axiocam 105 color; Zeiss) with integrated software (Zen Digital Imaging; Zeiss, Oberkochen, Germany).

### 4.2. Immunofluorescence

Only rats taken out from behavioral groups with the highest dose of morphine (1.5 µg) and naloxone (25.0 µg) were selected for this stage of the experiments (rats from Mc-5 (n = 5) and Mi-5 (n = 5) groups and rats from Nc-3 (n = 6) and Ni-3 (n = 6) groups), as well as additional rats from the control group in which only Es-VTA by 14 days was performed without injection (control group, n = 5). 

Immunofluorescent staining and imaging for *c-fos* expression (density of c-Fos positive cells) were performed according to a previous study [84]. The immunofluorescent procedure was described in detail previously [27,85,86].

All steps of the staining procedure were performed at room temperature and with the use of phosphate-buffered saline (PBS; pH 7.4), normal goat serum (NGS, Sigma-Aldrich; 5% NGS containing 0.3% Triton X-100), 0.5% bovine serum albumin (BSA, Sigma-Aldrich), and primary (*c-fos* mouse monoclonal IgG, Santa Cruz Biotechnology, Dallas, TX, USA at a dilution of 1:400 in PBS containing 0.3% TritonX-100 and 3% NGS) and secondary antibodies (CF543 Goat Anti-Mouse IgG, spectrally similar to Alexa Fluor 546; Invitrogen, Waltham, MA USA, at a dilution of 1:500).

Fluorescent images were taken using a microscope, Primo Star (Carl Zeiss MicroImaging GmbH, Oberkochen, Germany) (4 × 10 and 20 × 10 magnifications). The photos were white-label processed on a black background in grayscale ranging from 0 (black) to 255 (white) (Carl Zeiss Imaging Systems, Oberkochen, Germany, Axio Vision Rel. 4.9.1). The borders of brain structures were determined based on the atlas of rats [28] using contour traces derived from the templates. The software computed the determined area of the brain structures (i.e., thresholding procedure), where the optical density and size filters were calibrated to count white grains (>70% white) exceeding sizes of 15 pixels (15 µm^2^). Thus, the software excludes any remaining particles inconsistent with the size of a cell nucleus at a given magnification. Next, the density of c-Fos positive cells (c-Fos+) was computed as the number of counted nuclei converted to 1 mm^2^ of the surface area of the analyzed structure.

### 4.3. Data Analysis

The results are presented as box-and-whisker plots representing median values, first and third quartiles, and maximum/minimum values collapsed over the 4 anteriority levels. 

Before performing statistical analysis of the data, tests were conducted for the normality of the distribution and equality of variances. Data analyses were performed using SPSS and Gretl software. The Shapiro–Wilk distribution normality test was performed in each experimental group (including the analyses of behavioral and immunofluorescent data). Additionally, for each experimental group, calculations were performed using the Doornik–Hansen test, the Jarque–Bera statistic value test, and the Lilliefors test. To test the equality of variances, we used Levene’s median test. The datasets passed the tests for normal distribution and equal variances. The fixed-effect model was used for statistical analysis. 

The influence of OR activation manipulation in the PPN on VTA-controlled behavior was assessed based on the percentage change in the threshold frequency and the FR latency. The changes were compared with the pre-injection baseline and ACSF injection as a control (in the ipsilateral or contralateral PPN). Percent changes in the threshold after drug injection, calculated based on the control (baseline as ACSF injection), were analyzed and compared using analysis of variance (two-way ANOVA after pharmacological injection with division into hemispheres (contra-/ipsilateral) and different doses) and expressed as mean ± SEM. Two-way ANOVA with 2 parameters, (1) control ACSF injection vs. opioid injection and (2) comparison of different frequencies, was used to analyze differences during the RF latency(s).

Statistical analyses of the mean density of c-Fos positive cells (c-Fos+) in selected brain limbic and extrapyramidal structures were performed using two-way ANOVA (the factors were the type and hemisphere of injection).

The differences in means were further analyzed with Tukey’s HSD post hoc test (α = 0.05). The confidence level was 95%. A *p*-value of 0.05 was considered to be a significant effect.

## Figures and Tables

**Table 1:** Localization of the electrode tips in the ventral tegmental area (VTA) in all rats with unilateral VTA electrical stimulation (Es-VTA) and morphine and naloxone injection (experimental groups, n = 86) and in rats only with Es-VTA (baseline group, n = 5), and localization of the cannula tips in all rats of the experimental groups (n = 86) that received morphine (Mc-1, Mc-2, Mc-3, Mc-4, Mc-5 and Mi-1, Mi-2, Mi-3, Mi-4, Mi-5 groups; n = 50) and naloxone (Nc-1, Nc-2, Nc-3 and Ni-1, Ni-2, Ni-3 groups; n = 36) into the pedunculopontine tegmental nucleus (PPN) according to the rat brain atlas [28].

Experimental Groups		Distance from Bregma (mm)					
		VTA			PPN		
		Number of Stimulation Electrode Tips			Number of Cannula Injection Tips in Contra-/Ipsilateral Side		
		−4.80	−5.20	−5.30	−7.64	−7.80	−8.00
Rats with morphine injection (n = 50)	Mc-1	3	1	1	1	2	2
	Mi-1	4	1	-	4	1	-
	Mc-2	4	-	1	-	3	2
	Mi-2	5	-	-	1	1	3
	Mc-3	1	4	-	4	1	-
	Mi-3	5	-	-	3	2	-
	Mc-4	3	2	-	2	1	2
	Mi-4	2	-	3	2	-	3
	Mc-5	4	1	-	-	4	1
	Mi-5	1	3	1	-	4	1
Rats with naloxone injection (n = 36)	Nc-1	3	2	1	2	4	-
	Ni-1	4	2	-	5	1	-
	Nc-2	5	1	-	-	1	5
	Ni-2	6	-	-	-	2	4
	Nc-3	4	1	1	4	1	1
	Ni-3	2	1	3	-	4	2
Rats only with Es-VTA (n = 5)	baseline	3	1	1	-	-	-

n: the number of rats in each experimental group; Mc-1, Mc-2, Mc-3, Mc-4, Mc-5: rats that received different doses of morphine (0.25 µg/0.5 µL: Mc-1; 0.5 µg/0.5 µL: Mc-2; 1.0 µg/0.5 µL: Mc-3; 1.25 µg/0.5 µL: Mc-4; 1.5 µg/0.5 µL: Mc-5) into the contralateral PPN hemisphere; Mi-1, Mi-2, Mi-3, Mi-4, Mi-5: rats that received different doses of morphine (0.25 µg/0.5 µL: Mi-1; 0.5 µg/0.5 µL: Mi-2; 1.0 µg/0.5 µL: Mi-3; 1.25 µg/0.5 µL: Mi-4; 1.5 µg/0.5 µL: Mi-5) into the ipsilateral PPN hemisphere; Nc-1, Nc-2, Nc-3: rats received different doses of naloxone (2.5 µg/0.5 µL: Nc-1; 5.0 µg/0.5 µL: Nc-2; 25.0 µg/0.5 µL: Nc-3) into the contralateral PPN hemisphere; Ni-1, Ni-2, Ni-3: rats received different doses of naloxone (2.5 µg/0.5 µL: Ni-1; 5.0 µg/0.5 µL: Ni-2; 25.0 µg/0.5 µL: Ni-3) into the ipsilateral PPN hemisphere.

## Data Availability

The data presented in this study are available upon request from the corresponding author.

## Abbreviations

ACSF	artificial cerebrospinal fluid
AD	anterodorsal thalamic nucleus
AgRP	agouti-related peptide neurons
AH	anterior hypothalamic area
AM	anteromedial thalamic nucleus
Amg	amygdala
Arc	arcuate hypothalamic nucleus
AV	anteroventral thalamic nucleus
Acb	accumbens nucleus
AcbSh	accumbens nucleus, part shell
CG1	cingulate cortex, area 1
CG2	cingulate cortex, area 2
Cpu	caudate putamen
DA	dopamine
DM	dorsomedial hypothalamic nucleus
Es-VTA	electrical VTA-stimulation
FR	feeding response
GABA	gamma-aminobutyric acid
Glu	glutamate
HDB	nucleus of the horizontal limb of the diagonal band
Hip	hippocampus
IF	interfascicular nucleus
LH	lateral hypothalamic area
LHb	lateral habenular nucleus
LSD	lateral septal nucleus, dorsal part
LSI	lateral septal nucleus, intermediate part
LSV	lateral septal nucleus, ventral part
LTP	long-term potentiation
MD	mediodorsal thalamic nucleus
MHb	medial habenular nucleus
ML	mesolimbic system
MS	medial septal nucleus
OR	opioid receptors
PAG	periaqueductal gray
PBP	parabrachial pigmented nucleus of the VTA
PFC	prefrontal cortex
PN	paranigral nucleus of the VTA
PPN	pedunculopontine tegmental nucleus
PVN	paraventricular hypothalamic nucleus
RLi	rostral linear nucleus of the raphe
RMTg	rostromedial tegmental nucleus
RSA	retrosplenial agranular cortex
RSG	retrosplenial granular cortex
SN	substantia nigra
SNC	substantia nigra, compact part
SNL	substantia nigra, lateral part
SNR	substantia nigra
STN	subthalamic nucleus, reticular part
SO	supraoptic nucleus of the hypothalamus
VDB	nucleus of the vertical limb of the diagonal band
VMH	ventromedial hypothalamic nucleus
VP	ventral pallidum
vSNC	ventral substantia nigra pars compacta
VTA	ventral tegmental area
ZI	zona incerta
